# FAM188B Expression Is Critical for Cell Growth via FOXM1 Regulation in Lung Cancer

**DOI:** 10.3390/biomedicines8110465

**Published:** 2020-10-31

**Authors:** Young Eun Choi, Hamadi Madhi, HaEun Kim, Jeon-Soo Lee, Myung-Hee Kim, Yong-Nyun Kim, Sung-Ho Goh

**Affiliations:** 1 Research Institute, National Cancer Center, 323 Ilsan-ro, Goyang 10408, Korea; 75162@ncc.re.kr (Y.E.C.); black6218@gmail.com (H.K.); luceinaltis@ncc.re.kr (J.-S.L.); 2 Department of Anatomy, Yonsei University College of Medicine, Seoul 03722, Korea; hamadimadhi2013@gmail.com (H.M.); mhkim1@yuhs.ac (M.-H.K.); 3 Department of Experimental Medicine, McGill University, Canada 845 Rue Sherbrooke O, Montréal, QC H3A 0G4, Canada

**Keywords:** FAM188B, FOXM1, lung cancer, cell survival, deubiquitination

## Abstract

Although family with sequence similarity 188 member B (FAM188B) is known to be a member of a novel putative deubiquitinase family, its biological role has not been fully elucidated. Here, we demonstrate the oncogenic function of FAM188B via regulation of forkhead box M1 (FOXM1), another oncogenic transcription factor, in lung cancer cells. FAM188B knockdown induced the inhibition of cell growth along with the downregulation of mRNA and protein levels of FOXM1. FAM188B knockdown also resulted in downregulation of Survivin and cell cycle-related proteins, which are direct targets of FOXM1. Interestingly, FOXM1 co-immunoprecipitated with FAM188B, and the levels of FOXM1 ubiquitination increased with FAM188B knockdown but decreased with FAM188B overexpression. In addition, in vivo xenograft of FAM188B siRNA (siFAM188B) RNA-treated cells resulted in the retardation of tumor growth compared with that in the control. Furthermore, protein levels of FAM188B and FOXM1 were elevated in the human lung cancer tissues, and FAM188B expression was negatively correlated with the overall survival of lung cancer patients. These results indicate that FAM188B exerts its oncogenic effects by regulating FOXM1 deubiquitination and thus its stability. Therefore, FAM188B might be a potential therapeutic target to control lung cancer progression.

## 1. Introduction

Family with sequence similarity 188 member B (FAM188B), also known as MINDY-4, is an evolutionally distant member of FAM63A protein (MINDY-1), which acts as a putative deubiquitinase that cleaves Lys-48–linked polyubiquitin chain [1]. Accordingly, it is annotated as a “Probable Ubiquitin Carboxyl-Terminal Hydrolase” in the Genecards database [2], but the function of FAM188B has not been extensively elucidated yet. We reported the elevated expression of FAM188B in human colorectal cancer cells, and found that FAM188B knockdown results in the activation and accumulation of p53, along with an increase in cell death [3]. However, as FAM188B knockdown also induces death of p53-null HCT116 cells, FAM188B may employ other pathways to exert pro-survival effects independent of the p53 pathway.

Forkhead box M1 (FOXM1) is a transcription factor of forkhead box (Fox) protein superfamily and is critical for cell proliferation owing to its spatiotemporal expression during the cell cycle [4]. FOXM1 is detected in highly proliferative cells of regenerating tissues [5] and is upregulated in many types of human cancers, contributing to tumor development [6,7]. FOXM1 functions as a cell-cycle regulator by itself or by mediating nuclear translocation of β-catenin for the activation of the transcription of c-Myc and cyclin D1 [8,9]. FOXM1 is critical for tumor progression as it promotes angiogenesis, invasion, metastasis [10], response to DNA damage as well as resistance to therapeutics that cause genotoxicity [11,12].

FOXM1 is regulated at the transcriptional as well as post-translational level. Transcription factors such as c-Myc [13], E2F [14], and FOXM1 itself [15] bind directly to the FOXM1 promoter and activate its transcription [4]. Among the post-translational modification of FOXM1, phosphorylation [16] and SUMOylation [17] affect its transcriptional activity and/or cytoplasmic translocation. FOXM1 degradation can be regulated by both E3 ubiquitin ligase, such as APC/C-Cdh1 [18], and deubiquitinases, such as USP5, USP21, and OTUB1 in glioblastoma and breast cancers [9,19,20]. As FOXM1 is a transcription factor for cell-cycle regulation, targeting FOXM1 stability by regulation of its ubiquitination process could be a potential strategy for the inactivation of FOXM1.

Owing to the putative deubiquitinase activity of FAM188B [1] and decreased ubiquitination of p53 upon knockdown of FAM188B [3], FAM188B may also regulate ubiquitination of other proteins, such as FOXM1 and hence, its protein levels, leading to growth inhibition. To test this hypothesis, we examined the effects of FAM188B knockdown on FOXM1 levels and its ubiquitination along with cell growth in lung cancer cell lines. Here, we report a novel function of FAM188B in the regulation of FOXM1 deubiquitination. We demonstrate that FAM188B knockdown results in the inhibition of cell growth owing to the downregulation of FOXM1. FAM188B forms immunocomplexes with FOXM1 and regulates its ubiquitination levels. FAM188B expression is negatively correlated with the survival of lung cancer patients. These findings suggest that FAM188B exerts its oncogenic effects by regulating FOXM1. Hence, targeting FAM188B could be a potential strategy for controlling lung cancer progression.

## 2. Experimental Section

### 2.1. Datasets for Informatic Analyses of FAM188B in Lung Adenocarcinoma

For assessment of genetic alterations, mRNA expression, and survival of FAM188B, TCGA Lung adenocarcinoma Firehose dataset (*n* = 588) from cBioportal [21], TCGA lung (*n* = 187) and Lee lung (*n* = 138) datasets were selected from Oncomine database Research edition V4.5 (https://www.oncomine.org; Ann Arbor, MI, USA).

### 2.2. Cell Culture

A549, H1299, and PC9 lung adenocarcinoma cell lines were obtained from American Type Culture Collection (Manassas, VA, USA) and maintained in Roswell Park Memorial Institute 1640 medium (Corning Inc., Corning, Manassas, VA, USA) supplemented with 10% fetal bovine serum (Corning Inc., Manassas, VA, USA) and 1× penicillin streptomycin (Thermo Fisher Scientific, Carlsbad, CA, USA) at 37 °C and 5% CO_2_.

### 2.3. siRNA Transfection

A549, H1299, and PC9 cells (5.0 × 10^5^) were transfected with the FAM188B siRNA (siFAM188B) or non-specific control siRNA (NC siRNA) (Qiagen, Hilden, Germany; SI04333014) at a concentration of 5 nM for various times using Lipofectamine RNAiMAX (Thermo Fisher Scientific, Carlsbad, CA, USA). The FAM188B target sequence was 5′-CTG ACC ATT GAC ACC ACC CAA-3′. Transfected cells were grown at 37 °C and 5% CO_2_ and then harvested for experiments.

### 2.4. Cell Proliferation Assay

A549 and H1299 cells grown in 6-well plates were transfected with siFAM188B or NC siRNA. Cell proliferation was recorded from the beginning of siRNA transfection and evaluated using the Incucyte™ ZOOM live cell imaging system (Essen Bioscience, Ann Arbor, MI, USA), and the accompanying software was used to monitor and calculate cell confluency in non-overlapping regions of each well.

### 2.5. Cell-Cycle Analysis

A total of 5.0 × 10^5^ A549 and H1299 cells were transfected with siFAM188B or NC siRNA for 72 h. Cells were trypsinized, collected by centrifugation, and resuspended in phosphate-buffered saline (PBS) before fixing in 70% ethanol. After incubation at 4 °C for at least 3 h, the cells were centrifuged and then incubated with 5 μg/mL propidium iodide and 0.1 mg/mL RNase A for 30 min before analysis on a FACS-LSR Fortessa flow cytometer (BD Biosciences, San Jose, CA, USA).

### 2.6. Apoptosis Analysis (Annexin V Assay)

A549 and H1299 cells (8.0 × 10^4^) in 6-well plates were transfected with siFAM188B or NC siRNA. The cells were stained by FITC Annexin V Apoptosis Detection Kit I (556547, BD Biosciences, San Diego, CA, USA) and were analyzed with FACSVerse.

### 2.7. Geneset Enrichment and Protein–Protein Interaction Analyses

Differentially expressed genes with log fold change >1.5 or <−1.5 and *p*-value < 0.001 between negative control and FAM188B knockdown A549 cells were selected (Table S1). Selected gene lists were queried for their enrichment in gene sets in Enrichr database [22]. Enriched gene lists were subjected to predict protein–protein interactions from String-DB [23].

### 2.8. Reverse Transcription Quantitative Real-Time PCR

Total RNA was isolated with the RNeasy kit (Qiagen) according to the manufacturer’s protocol. The PrimeScript 1st Strand cDNA Synthesis kit (Takara Bio, Otsu, Japan) was used to obtain cDNA. Primers used in this study are listed in Table S2. Amplification was performed (*n* = 3) on a real-time PCR system (Roche, Basel, Switzerland; LC480) as described in a previous report [3].

### 2.9. Immunoblot Analysis

For analysis of protein expression, siRNA-transfected cells were harvested and lysed in M-PER buffer (Thermo Fisher Scientific, Waltham, MA, USA) containing protease inhibitor (Roche) for 30 min at 4 °C. To analyze protein subcellular localization, cell pellets were lysed with nuclear fraction lysis buffer containing protease inhibitor (Abcam, Cambridge, MA, USA) for 1 h at 4 °C according to the manufacturer’s protocol. Cytoplasmic proteins were obtained by lysing cells in Buffer A composed of 10 mM HEPES, 1.5 mM MgCl_2_, 10 mM KCl, 0.5 mM dithiothreitol (DTT), and 0.05% Nonidet P-40 (pH 7.9, while nuclear proteins were obtained using Buffer B composed of 5 mM HEPES, 1.5 mM MgCl_2_, 0.2 mM EDTA, 0.5 mM DTT, and 26% glycerol (*v*/*v*) (pH 7.9. Protein concentration was determined using a bicinchoninic acid protein assay kit (Cat. 23225, Thermo Fisher Scientific, Carlsbad, CA, USA), and equal amounts of protein were boiled and separated by sodium dodecyl sulfate–polyacrylamide gel electrophoresis and transferred to a polyvinylidene difluoride membrane that was blocked with 3% skim milk in Tris-buffered saline with Tween 20 and probed overnight at 4 °C with primary antibodies against FAM188B (AbFrontier, Seoul, Korea); FLAG-M2 (F1804) from Sigma-Aldrich (Saint Louis, MO, USA); β-catenin (sc-7963), FOXM1 (sc-271746), Bcl-XL (sc-7195), cyclin E1 (sc-481), CDK2 (sc-163), GAPDH (sc-25778), β-actin (sc-47778) and α-Tubulin(sc-8035) (all from Santa Cruz Biotechnology, Santa Cruz, CA, USA); and c-Myc (5605S), cyclin D1 (2978S), phosphor-BAD (4366S), XIAP (2045S), Survivin (2808S), Aurora kinase B (3094S), cyclin B1 (4138S) and PARP (9542S) (Cell Signaling Technology, Danvers, MA, USA). The membrane was then incubated with an appropriate horseradish peroxidase-conjugated secondary antibody for 1 h at room temperature, and protein bands were visualized using and enhanced chemiluminescence kit (Thermo Fisher Scientific, Carlsbad, CA, USA).

### 2.10. Immunohistochemistry

Formaldehyde-fixed, paraffin-embedded tissue sections were used for immunohistochemistry with an anti-FAM188B antibody (HPA030130, Atlas Antibodies AB, Stockholm, Sweden) and a Discovery XT automated slide stainer (Ventana Medical Systems, Tucson, AZ, USA). Serial sections incubated with normal IgG instead of primary antibody were used as a negative control.

### 2.11. Immunocytochemistry

Cells cultured on coverslips were transfected with siFAM188B or NC siRNA for 48 h and fixed with 4% paraformaldehyde. After permeabilization, cells were incubated with anti-β-catenin and anti-FOXM1 antibodies diluted 1:50 followed by secondary antibody labeled with fluorescent dye. Nuclei were stained with Hoechst33342. After washing with PBS, cells were viewed under a microscope and images were acquired using Axiovision software (Carl Zeiss, Oberkochen, Germany).

### 2.12. Co-Immunoprecipitation Analysis

For analysis of protein interactions, 4 ug of FLAG-tagged expression construct of FAM188B or FOXM1B plasmid was transfected into HEK-293, H1299, and PC9 cells using Lipofectamine 2000 according to the manufacturer’s protocol (Thermo Fisher Scientific, Carlsbad, CA, USA) and incubated for 48 h at 37 °C in 5% CO_2_ incubator. Transfected cells were harvested and lysed in M-PER buffer (Thermo Fisher Scientific, Carlsbad, CA, USA) containing Complete mini protease inhibitor cocktail (Cat. 04 693 124 001, Roche, Diagnostics, Mannheim, Germany) for 30 min at 4 °C. Five hundred microgram of cell lysate was used for each immunoprecipitation with anti-FLAG-M2 affinity agarose beads (A-2220, Sigma-Aldrich, St. Louis, MO, USA). The mixture of cell lysate and anti-FLAG-M2 agarose was incubated at 4 °C for 16 h and then washed according to the manufacturer’s protocol. Immunoprecipitated proteins resolved on SDS-PAGE gel and transferred to Immobilon-P PVDF-membrane (Cat. IPVH 000 10, Millipore, MA, USA). Interacting protein was detected by immunoblotting as described above.

### 2.13. In Vivo Analysis of FAM188B Knockdown

A549 cells were transfected with siFAM188B or NC siRNA. Transfected cells (2.0 × 10^6^) were subcutaneously injected in BALB/c nude mice (*n* = 10). The experiment was approved by Institutional Animal Care and Use Committee in National Cancer Center of Korea (NCC-16-231).

### 2.14. Statistical Analysis

Differences between groups were evaluated with the chi-square test and Student’s *t*-test. *p*-values < 0.05 were considered statistically significant. Variation was represented as standard deviation.

## 3. Results

### 3.1. Effects of FAM188B Knockdown on Cell Growth

**Figure 1:**
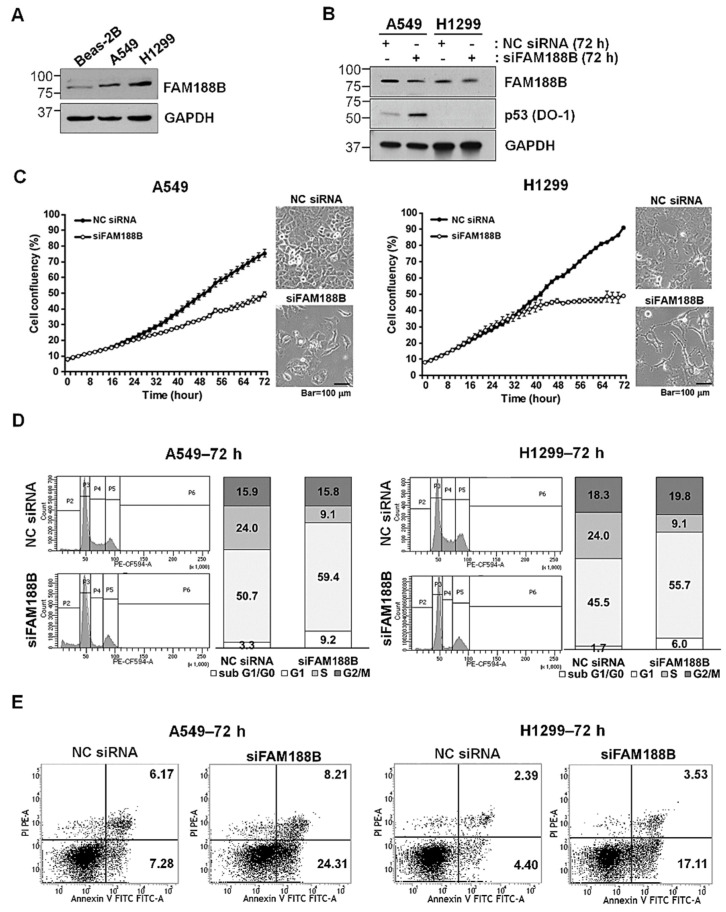
Effects of family with sequence similarity 188 member B (*FAM188B*) knockdown on proliferation and cell cycle in lung cancer cell lines. (**A**) Equal amount of cellular proteins from Beas-B2, a normal lung cell line, and A549 and H1299 lung cancer cell lines was processed for immunoblot analysis using indicated antibodies. GAPDH was used as a loading control. (**B**–**E**) A549 and H1299 cells were transfected either with non-specific control siRNA (NC siRNA) or siRNA specific for *FAM188B* (siFAM188B) at indicated times. Cells were then either processed for immunoblot analysis using indicated antibodies (**B**), cell growth analysis using Incucyte™ (**C**), cell-cycle analysis by flow cytometry (**D**), or apoptosis assay by flow cytometry of annexin-V/PI stained cells (**E**). Annexin-V-positive populations were considered as apoptotic populations. Scale bar; 100 μm. These experiments were performed three times with comparable results.

To investigate whether FAM188B is involved in lung cancer progression, FAM188B levels in lung cancer cell lines as well as in Beas-2B—a normal lung epithelial cell line—were examined. FAM188B protein levels were higher in the lung cancer cells than those in Beas-2B cells (Figure 1A). To test whether *FAM188B* knockdown affects cell growth, A549 cells with wild-type *p53* expression and *p53*-null H1299 cells [24] were transfected with FAM188B siRNA (siFAM188B) for 72 h (Figure 1B). Cell growth imaging and cell confluency monitoring with Incucyte™ image analysis (Figure 1C) revealed that compared with the negative control (NC) siRNA treatment, FAM188B knockdown retarded cell growth in both cell lines. These results indicate that FAM188B expression is important for cell growth. The inhibition of cell growth can be attributed to altered progression of cell cycle or cell death. To examine these possibilities, we performed cell-cycle analysis of siFAM188-treated cells by flow cytometry. As illustrated in Figure 1D, the sub-G0/G1 fraction increased whereas S- and G2-phase populations decreased between 48 and 72 h in A549 cells upon *FAM188B* knockdown. In H1299 cells, *FAM188B* knockdown decreased the S-phase population but increased G2-phase population with no detectable sub-G0/G1 fraction (Figure 1D and Figure S1A). These results indicate that *FAM188B* knockdown retarded the G1/S phase transition with little cell death. Apoptosis induced by *FAM188B* knockdown was examined by the flow cytometry analysis of annexin-V/PI stained cells. Apoptotic populations increased in a time-dependent manner in the siFAM188B-treated cells (Figure 1E and Figure S1B). Collectively, these data suggest that FAM188 is important for cell-cycle regulation and survival.

### 3.2. Altered Transcriptome Profiling Induced by FAM188B Knockdown

**Figure 2:**
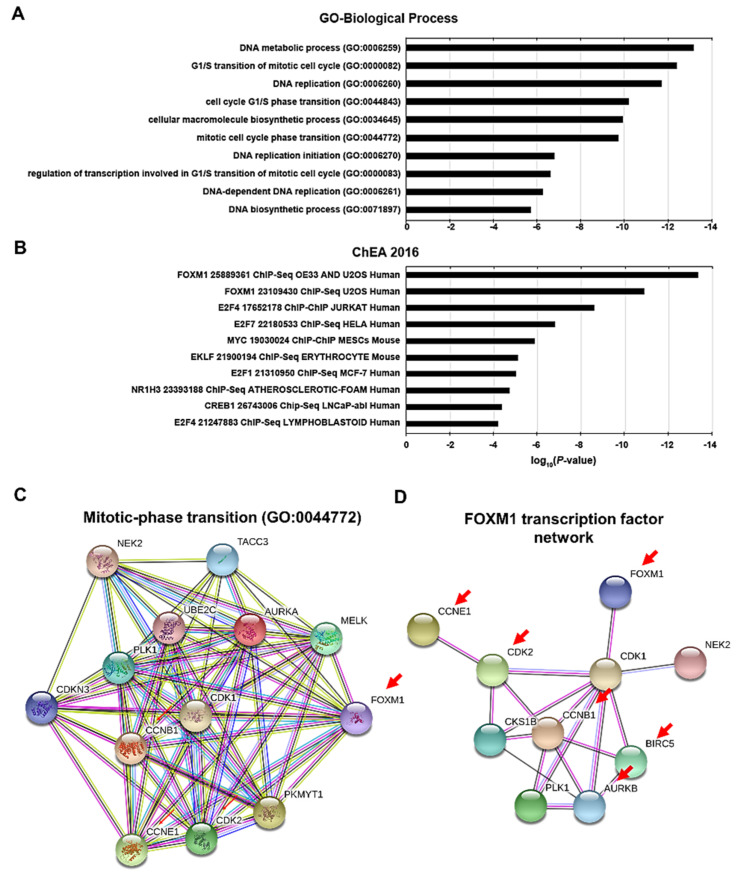
Altered transcription profiling and signaling pathways induced by *FAM188B* knockdown (**A**,**B**) A549 cells were transfected either with NC siRNA or siFAM188B for 48 h, followed by mRNA purification and RNAseq analysis. Downregulated genes by siFAM188B from RNAseq data were subjected to gene set enrichment analyses for gene ontology (GO)-biological process (**A**) and ChIP-X Enrichment Analysis (ChEA 2016) gene sets (**B**). Predictions of protein–protein interactions of downregulated genes categorized into forkhead box M1 (FOXM1) transcription factor network (**C**) and mitotic-phase transition by String-DB database (**D**).

As *FAM188B* knockdown resulted in cell growth inhibition owing to cell-cycle changes and apoptosis in A549 cells, we analyzed changes in transcriptome profiles upon *FAM188B* knockdown in A549 cells with RNAseq data for the enriched biological pathways using gene-set enrichment analyses with Enrichr database [22]. We observed that the genes or proteins with a decreased expression upon *FAM188B* knockdown were enriched in the categories of DNA metabolic process and replication gene sets, which are linked to the cell cycle in gene ontology (GO)-biological process (Figure 2A). Genes of which expression is decreased by *FAM188B* knockdown were analyzed to be regulated by FOXM1, c-MYC, and E2F transcription factor in based on ChIP-X Enrichment Analysis (ChEA 2016) (Figure 2B). Clustering analysis of the genes of which expression is decreased using String-DB database [23] demonstrated a strong connection between them (Figure 2C,D). Among the protein–protein interactions generated by these genes, the cluster classified by Mitotic-phase transition of GO-biological process showed multiple interactions with crucial proteins involved in mitotic phase transition (Figure 2C). Intriguingly, *FOXM1* gene, known as an oncogene, was filtered as gene with statistically significant reduced expression (*p* < 0.001) (Table S1). Furthermore, FOXM1 links to the CDK1, which interacts with a set of genes in the clusters that were downregulated by *FAM188B* knockdown (Figure 2D). These results suggest that *FAM188B* knockdown decreases expression of transcription factors that are involved in cell-cycle progression and survival, such as FOXM1 and MYC, and their downregulation might play a role in the inhibition of growth induced by *FAM188B* knockdown.

**Figure 3:**
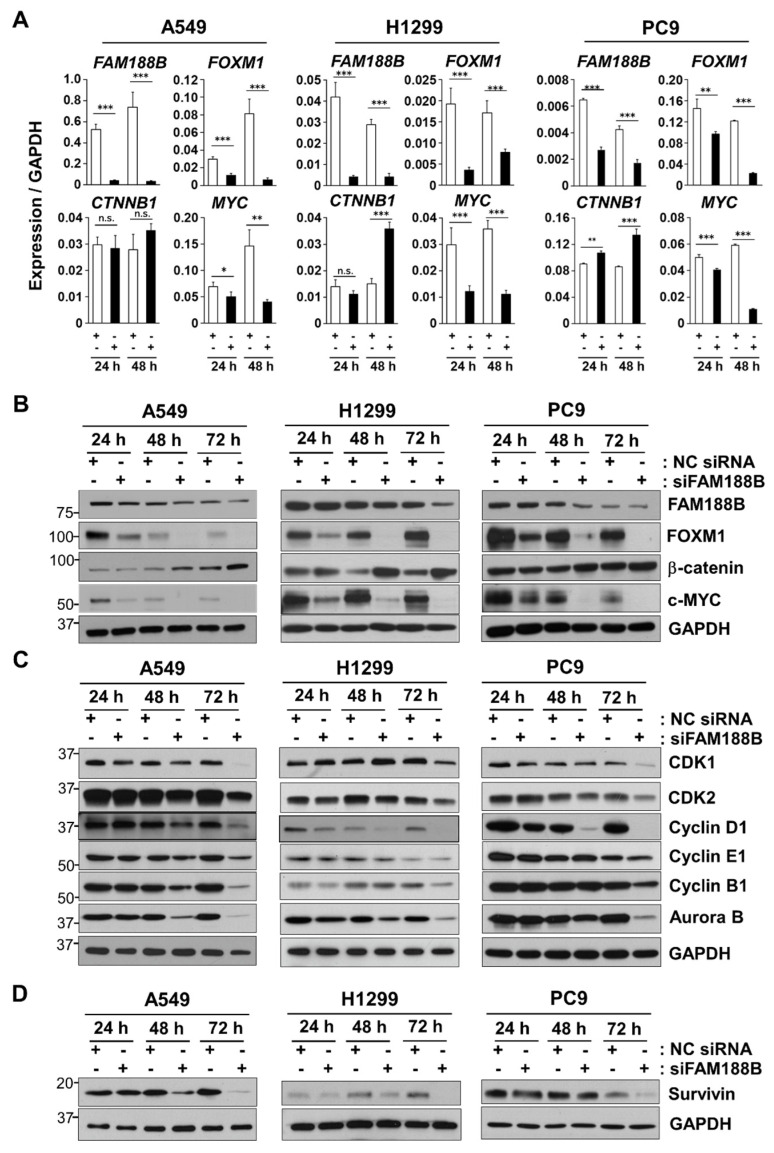
Effects of *FAM188B* knockdown on expression levels of oncogenic transcription factors, cell cycle-regulatory proteins and cell survival proteins (**A**–**D**) A549, H1299, and PC9 cells were transfected either with NC siRNA or siFAM188B for indicted times. Cells were then processed either for qPCR analysis of *FAM188B*, *FOXM1*, *CTNNB1* (β-catenin gene), and *c-MYC*, (**A**) or for immunoblot analysis using indicated antibodies (**B**–**D**). GAPDH was used as a control for qPCR and immunoblot analysis. These experiments were conducted two times independently with comparable results. The levels of proteins were quantified by a densitometry and normalized to GAPDH (Figure S7). Error bars represent standard deviations of the mean of three measurements (* *p* < 0.05, ** *p* < 0.01, *** *p* < 0.001, and n.s. for not significant).

Next, we performed qRT-PCR to validate the change in the expression of genes upon *FAM188B* knockdown. Owing to the over-expression of β-catenin, which has been associated with tumor progression [25] and the involvement of FOXM1 in the translocation of β-catenin to the nucleus [8], the effect of *FAM188B* knockdown on β-catenin expression was tested. Consistent with our RNAseq data, *FAM188B* knockdown decreased the expression of both *FOXM1* and *MYC* in A549, H1299, and PC9 cells (Figure 3A). However, it upregulated *CTNNB1* (β-catenin gene) mRNA expression in all three cell lines. These results indicate that FAM188B affects the expression of FOXM1, β-catenin, and MYC, which are important factors for the regulation of cell growth. Regulation of these molecules by FAM188B was not selective to A549 as similar results were observed in H1299 and PC9 cells (Figure 3A). Consistent with mRNA expression data in Figure 3A, *FAM188B* knockdown decreased protein levels of FOXM1 and c-MYC, but not β-catenin (Figure 3B). We investigated whether siFAM188B affects the expression of cell cycle-regulating proteins. *FAM188B* knockdown resulted in the downregulation of various cell cycle-associated proteins, such as CDK1, CDK2, cyclin D1, cyclin E1, cyclin B1, and Aurora kinase B (Figure 3C). Although a variation was observed dependent on cell lines, siFAM188B resulted in decreased levels of anti-apoptosis proteins, such as Survivin (Figure 3D). As FOXM1 regulates G1/S and G2/M transition and cell survival via the expression of CDKs, cyclins, Aurora kinase B, and Survivin [5], downregulation of FOXM1 induced by siFAM188B might be responsible for a decrease in the expression of these proteins, thereby leading to growth inhibition.

### 3.3. FAM188B Modulates Intracellular Localization of FOXM1 and β-Catenin

**Figure 4:**
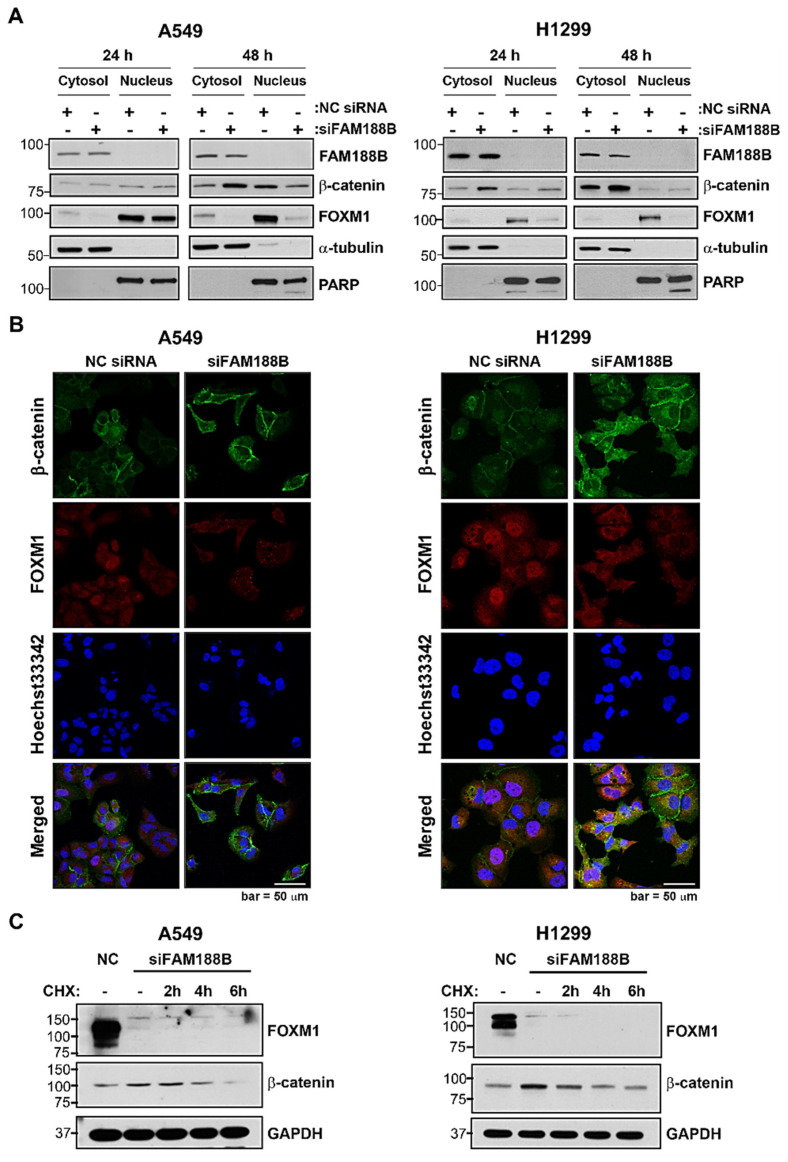
Effects of *FAM188B* knockdown on expression levels and nuclear translocation of FOXM1 and β-catenin (**A**) A549 and H1299 cells were transfected either with NC siRNA or siFAM188B for indicted times. Cells were then fractionated into cytosolic and nuclear fractions, followed by immunoblot analysis using indicated antibodies. PARP and α-tubulin were used as a cytoplasmic and nuclear marker, respectively. (**B**) Cells treated as in (**A**) for 48 h were stained for β-catenin and FOXM1 and fluorescence images were captured using confocal microscope. Nuclei were stained with Hoechst33342. Scale Bar: 50 μm (**C**) Cells were treated as in (**A**) for 48 h and were treated with cycloheximide (CHX) for 2–6 h before harvest. Total cellular proteins were analyzed by immunoblot using indicated antibodies. These experiments were performed two times independently with similar results.

Here, *FAM188B* knockdown downregulated the expression of FOXM1, c-Myc, and cyclin D1, but increased β-catenin expression (Figure 3A,B). FOXM1 functions as a cell-cycle regulator by mediating the nuclear translocation of β-catenin to activate c-Myc and cyclin D1 [4,8]. Although the expression of β-catenin increased, its nuclear translocation was inhibited owing to FOXM1 downregulation in the siFAM188B-transfected cells. We compared the protein expression of β-catenin and FOXM1 in the nuclear and cytoplasmic fractions after the transfection of A549 and H1299 cells with that of siFAM188B. As expected, cytoplasmic β-catenin increased but nuclear β-catenin decreased upon *FAM188B* knockdown in both A549 and H1299 cells (Figure 4A). FOXM1 was present primarily in the nuclear fraction. However, it disappeared from both cytoplasmic and nuclear fractions in the siFAM188B-transfected cells due to FOXM1 downregulation (Figure 4A). When their subcellular localization was examined using immunofluorescence analysis, the overall intensity of β-catenin staining became stronger whereas that of FOXM1 was weaker in the siFAM188B-treated A549 cells compared with NC siRNA (Figure 4B), which is consistent with the immunoblot data in Figure 3B. FOXM1 was primarily stained in the nucleus and β-catenin was observed in the cytoplasm and cell membrane in the NC siRNA-treated cells. Only a small amount of β-catenin was detected in the nucleus. However, β-catenin expression became stronger in siFAM188B-treated cells (Figure 4B), indicating that *FAM188B* knockdown involves in the regulation β-catenin level, but not its nuclear translocation. The immunofluorescence data are consistent with the cell fractionation data (Figure 4A), which were also observed in the H1299 cells (Figure 4B). To test whether β-catenin upregulation induced by *FAM188B* knockdown is due to the de novo synthesis of β-catenin, cycloheximide (CHX) chase assay was performed. The level of β-catenin was increased by siFAM188B treatment, but when de novo protein synthesis was inhibited by CHX, β-catenin levels decreased in a time-dependent manner in the siFAM188B-treated cells (Figure 4C). This result indicates that FAM188B is involved in the regulation of β-catenin protein level via de novo synthesis as well as regulation of its half-life.

### 3.4. FAM188B Interacts with FOXM1 and Regulates Stability via Deubiquitination

**Figure 5:**
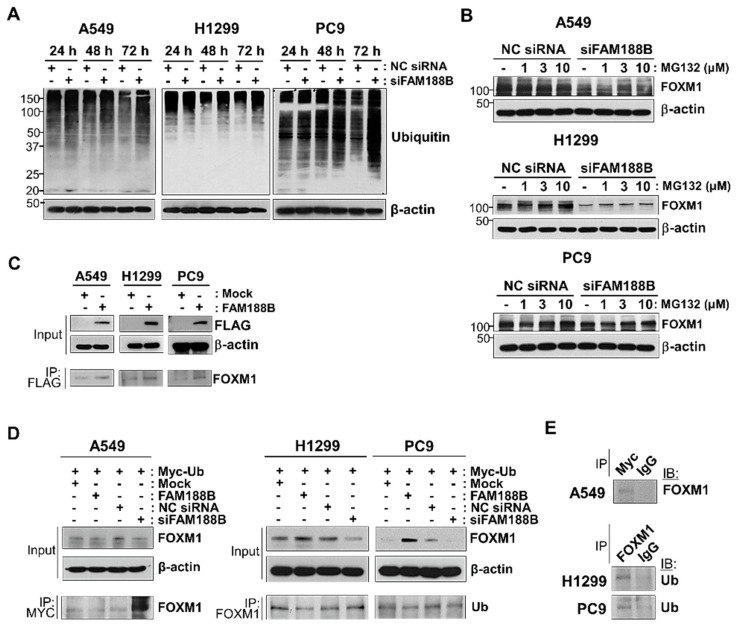
Effects of FAM188B on ubiquitination levels of both total proteins and FOXM1. (**A**) Total protein ubiquitination by *FAM188B* knockdown. A549, H1299, and PC9 cells were transfected either with NC siRNA or siFAM188B for indicted times. Cells were then processed for immunoblot analysis using indicated antibodies. The levels of proteins were quantified by a densitometry and normalized to GAPDH (Figure S8). (**B**) Recovery of downregulated FOXM1 by MG132 treatment in siFAM188B-treated lung cancer cells. Cells were transfected with siRNA for 24 h and then treated with MG132 with 1, 3, and 10 uM for 16 h. Level of FOXM1 was detected by immunoblot. (**C**) Detection of physical interaction of FOXM1 with β-catenin in lung cancer cells. Cells were transfected with FLAG-tagged FAM188B (FAM188B) for 72 h and were subjected to immunoprecipitation (IP) assay using anti-flag antibodies. Equal amounts of cell lysates (Input) and immunocomplexes were analyzed by immunoblot using indicated antibodies. (**D**) Cells were co-transfected with Myc-tagged ubiquitin (Myc-Ub) and either with mock, FAM188B, NC siRNA, or siFAM188B ubiquitin followed by IP using either anti-MYC antibody (in A549 cells) or anti-FOXM1 antibodies (in H1299 and PC9). Equal amount of cell lysates (Input) and immunocomplexes were analyzed by immunoblot using indicated antibodies. The levels of proteins were quantified by a densitometry and normalized to GAPDH (Figure S8). (**E**) Cells were transfected with Myc-Ub, followed by IP using either anti-MYC or anti-FOXM1 antibodies. Non-specific IgG (IgG) was used for IP control antibody. Immunocomplexes were analyzed by immunoblot using indicated antibodies. These experiments were performed two times independently with similar results.

FAM188B is a distant member of the novel deubiquitinase family FAM63A [1]. We first examined whether *FAM188B* knockdown affects the overall ubiquitination level. In three lung cancer cell lines, compared with that in the control treatment, the ubiquitination of total cellular proteins remained unchanged at 24 h but increased at 48 h and 72 h after siFAM188B treatment (Figure 5A). As FOXM1 was downregulated owing to *FAM188B* silencing (Figure 3), the turnover of FOXM1 protein might be mediated by proteasomal degradation. To evaluate this, cells were treated with MG132, a proteasome inhibitor, at 24 h after siRNA transfection. FAM188B-induced FOXM1 downregulation was reversed by MG132 treatment in a dose-dependent manner in all three cell lines (Figure 5B). These results indicate that proteasomal degradation is involved in the downregulation of FOXM1 upon *FAM188B* knockdown. To examine whether FAM188B forms complexes with FOXM1, A549, H1299, and PC9, cells were transfected with FLAG-tagged FAM188B, followed by analysis with a co-immunoprecipitation assay using anti-Flag antibodies. FLAG-tagged FAM188B expression in each cell line was verified (Figure 5C). FOXM1 was detected in the FAM188B immunocomplexes in A549, H1299, and PC9 cells (Figure 5C).

To investigate whether FOXM1 ubiquitination is regulated by FAM188B expression, cells transfected with MYC-tagged ubiquitin and FAM188B were either overexpressed or silenced, followed by immunoprecipitation of either MYC-tagged ubiquitin (A549 cells) or FOXM1 (H1299 and PC9 cells). In A549 cells, levels of ubiquitinated FOXM1 were lower with FAM188B overexpression than in those with mock expression (Figure 5D). However, the levels of ubiquitinated FOXM1 were significantly higher in siFAM188B-treated cells than those in NC siRNA (Figure 5D), indicating that FOXM1 ubiquitination is regulated by FAM188B expression. FAM188B overexpression increased and its knockdown resulted in decreased FOXM1 in H1299 and PC9 cells (Figure 5D). Levels of ubiquitinated FOXM1 in the immune complexes decreased with FAM188B overexpression but they increased marginally with *FAM188B* knockdown (Figure 5D). FOXM1 and its ubiquitination were scarcely detected in the immunocomplexes of control IgG antibodies in the three cell lines (Figure 5E). These results indicate that FAM188B expression regulates FOXM1 levels via ubiquitination.

### 3.5. FAM188B Downregulation Inhibits Tumor Growth In Vivo

**Figure 6:**
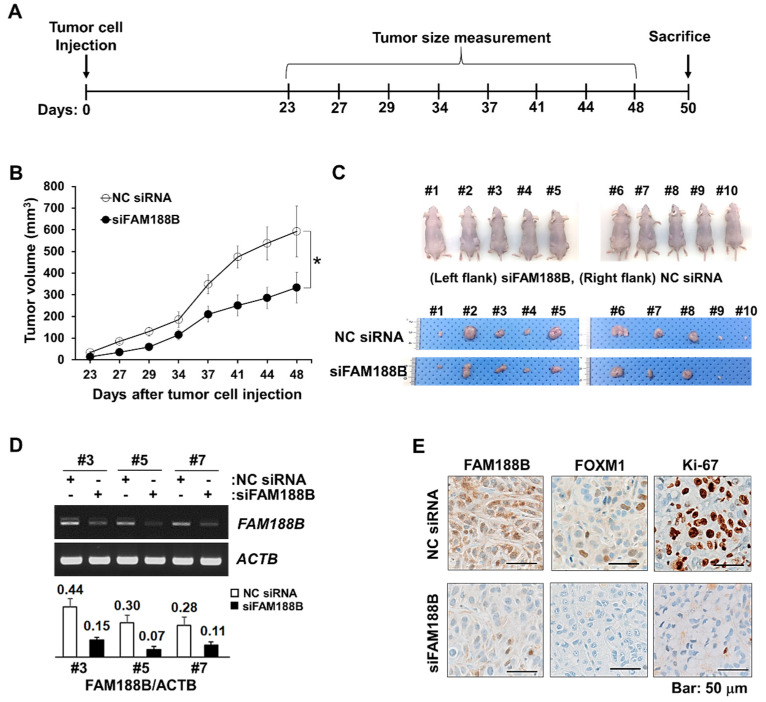
Effects of *FAM188B* knockdown on tumor growth mouse xenograft model. (**A**) Schematic procedure of in vivo experiments. A549 cells were transfected either with NC siRNA or siFAM188B for 48 h and cells were injected subcutaneously. (**B**) Two sets of experiments were shown, and the sizes of tumors were measured on indicated days (5 mice in each group). Error bars represent standard deviations. * *p* < 0.05 (**C**) The mice were sacrificed on day 50 and images of isolated tumors were captured. (**D**) Some tumors were processed for RT-PCR of *FAM188B* and levels of *FAM188B* were normalized by *ACTB* (β-actin gene) as presented in the bar plot (□: NC siRNA, ■: siFAM188B). (**E**) Immunohistochemistry of FAM188B, FOXM1, and Ki-67 of representative tumor tissue (#7). Scale bar; 50 μm. These experiments were performed two independent times with comparable results.

To evaluate whether FAM188B could be a potential target for controlling tumor growth, A549 cells that were transfected with either NC siRNA or siFAM188B for 48 h were subcutaneously injected in mice (*n* = 10) and tumor growth was monitored as illustrated in Figure 6A. Compared with the NC siRNA-treatment group, the siFAM188B-treated group showed significantly retarded tumor growth rate. Decreased tumor growth was observed after 27 days following xenograft (Figure 6B). The tumors were isolated from the euthanized mice on day 48 (Figure 6C). As shown in Figure 6C, the sizes of tumors were smaller in the siFAM188B-treatment group than those in the control group, which is consistent with the reduced tumor volumes measured during the observation period (Figure 6B). Isolated tumors were then processed for total RNA extraction and immunohistochemistry. The expression of *FAM188B* mRNA was decreased in the siFAM188B-treatment group as observed by RT-PCR (Figure 6D). Immunohistochemistry analysis demonstrated that protein levels of FOXM1 and Ki-67, a proliferation marker, were down-regulated with *FAM188B* knockdown in the tumor tissue (Figure 6E). These results provide compelling evidence that *FAM188B* knockdown inhibits tumor growth in vivo.

### 3.6. Clinical Relevance of FAM188B Expression in NSCLC Patients

**Figure 7:**
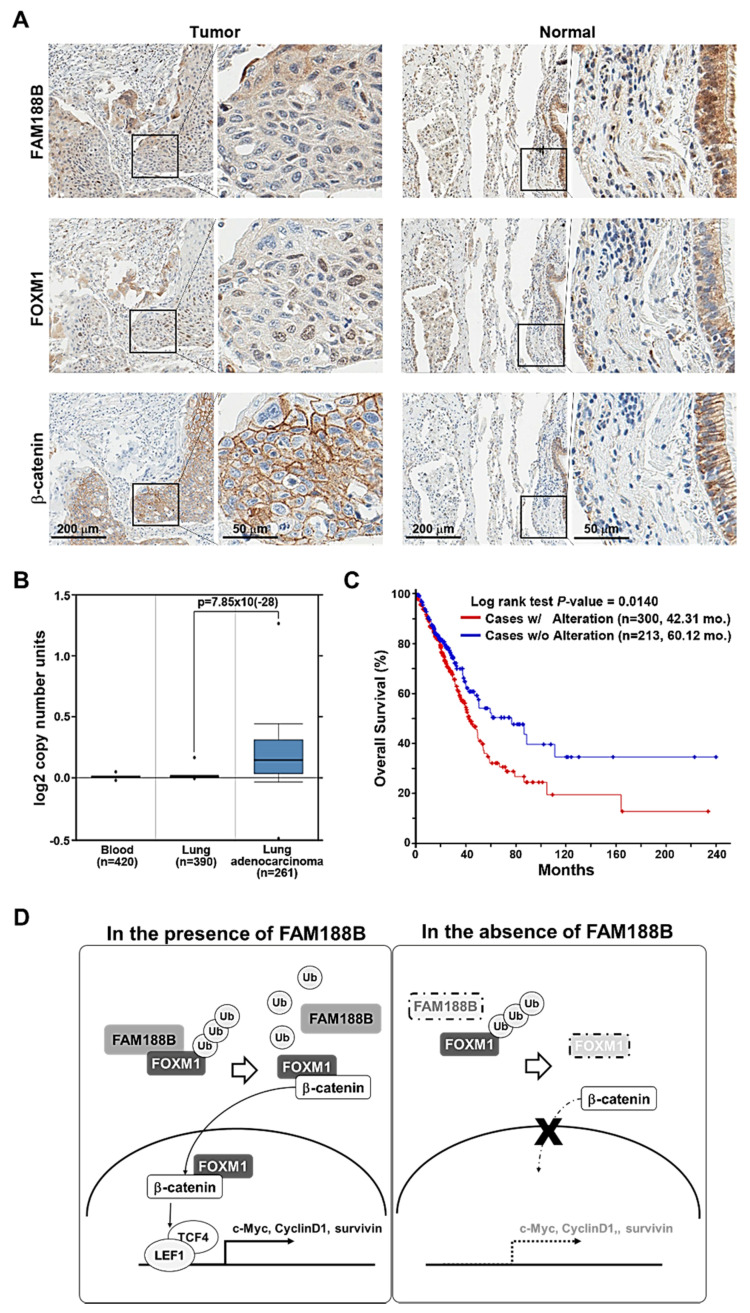
Clinical relevance of FAM188B expression in lung adenocarcinoma patients and a proposed model for an oncogenic role of FAM188B via FOXM1 regulation. (**A**) Immunohistochemical analysis of levels of FAM188B, FOXM1, and β-catenin in lung cancer tissues and normal lung tissues. Images of in each set were taken at 10× (left panel, scale bar; 200 μm) and boxed areas were magnified at 40× (right panel, scale bar; 50 μm). (**B**) Oncomine survey of significantly different copy number unit number between normal vs. cancer in TCGA lung 2 (*p* = 7.85 × 10^−28^). (**C**) Overall survival curves for lung cancer patients with (red line) or without (blue line) *FAM188B* alteration from TCGA Firehose LUAD (*n* = 586) dataset (*p* = 0.0140). (**D**) Schematic proposed model of FAM188B in association with FOXM1 regulation.

To explore the clinical significance of *FAM188B*, we examined FAM188B protein expression in human lung cancer tissue with microarray analysis from 50 patients, and 46 out of 50 tumor tissues were kept intact for interpretation. Immunohistochemical analyses of tumor tissues demonstrated that FAM188B expression was predominantly enhanced in 28 out of 46 tumor tissues (60.9%) (Figure 7A, top panel, Tables S3 and S4). FAM188B was primarily localized in the cytoplasm but certain cells demonstrated immunopositivity in the nucleus. FOXM1 staining was positive in 38 out of 46 tumor tissues (82.6%) and was observed primarily in the nucleus (Figure 7A, middle panel). β-catenin stained positive in all tumor tissues (100%) and was distributed in the cell membrane and the cytoplasm (Figure 7A, lower panel). FAM188B, FOXM1, and β-catenin primarily stained negative in the normal tissues (Figure 7A), indicating that these proteins are upregulated in the lung tumor tissues. Analysis of the correlation between the expression of FAM188B and FOXM1 in lung tumor tissues resulted in the correlation coefficient of r^2^ = 0.1079 (Figure S4). To determine the amplification of *FAM188B* in clinical samples, we used the Oncomine database V4.5 (https://www.oncomine.org). 

The DNA copy number of *FAM188B* was significantly increased in lung adenocarcinoma and squamous cell lung carcinoma compared with that in normal blood and lungs in TCGA lung 2 (https://www.oncomine.org/resource/ui/component/dataset.html?component=d:156636501) and Weiss lung cancer dataset (https://www.oncomine.org/resource/ui/component/dataset.html?component=d:156636653) [26] with a threshold of *p* < 0.0001 (Figure 7B and Figure S5). Moreover, the analysis of the lung cancer patients dataset from cBioPortal [21] demonstrated that the median overall survival was 42.31 months with *FAM188B* alteration (*n* = 300), whereas it was 60.12 months without *FAM188B* alteration (*n* = 213) (*p* = 0.0140) (Figure 7C). Further, median disease-/progression-free survival was 30.98 months (*n* = 252) and 47.63 months (*n* = 185), with and without *FAM188B* alteration, respectively (*p* = 0.0784) (Figure S6). These data indicate that FAM188B expression is upregulated in lung cancer patients and that alteration in *FAM188B* is negatively correlated with patient survival.

Taken together, we propose that FAM188B expression is critical for lung cancer progression. FAM188B exerts its effects by the regulation of FOXM1 via the inhibition of FOXM1 ubiquitination and degradation, which leads to FOXM1 accumulation (Figure 7D). The upregulated FOXM1 induces transcription of genes involved in cell proliferation, resulting in tumor growth. Therefore, FAM188B could be a potential target for controlling lung cancer progression.

## 4. Discussion

Although FAM188B has been reported as a putative deubiquitinase, its biological function has not been explored. Here, we demonstrate that FAM188B influences cell growth by regulating FOXM1 protein levels in lung cancer cell lines. FAM188B expression in the lung cancer patient tissues and its clinical relevance with survival was also illustrated.

The regulation of the activity and stability of certain proteins can be accomplished by balanced control between ubiquitination and deubiquitination. Ubiquitination/deubiquitination is especially important for the proteins involved in the regulation of cell cycle, proliferation, and survival. Dysregulation of their stability or activity can initiate tumors. A subset of deubiquitinases (DUBs) is involved in regulating the stability of key oncogenic or tumor suppressor proteins and is garnering interest as therapeutic targets for the influence on oncogenic signaling pathways [27]. FAM188B was introduced as a novel putative DUB [1] and is suggested to have oncogenic properties [3]. Recently, it was reported that risk allele A of SNP rs2041570 is associated with low level of FAM188B from the genome-wide association study of refractory celiac disease type II [28]. However, the biological function has not been extensively elucidated yet.

FOXM1 leads to tumorigenesis resulting in various cancers including breast, colorectal, gastric, hepatoma, and lung cancers [29]. The expression of FOXM1 can also lead to a wide range of oncogenic abnormalities including angiogenesis, cell proliferation, migration, metastasis, and drug resistance [19]. FOXM1 expression is regulated at transcriptional, post-transcriptional, and post-translational levels [4]. For post-translational modification, several DUBs can regulate FOXM1 stability [9,19,20]. We suggested that FAM188B is a putative DUB for FOXM1 regulation, which has been uncharacterized so far. FAM188B regulates FOXM1 both at transcriptional and post-translational levels. Decreased *FOXM1* mRNA levels were observed from transcriptome analysis (Figure 2) and qPCR data (Figure 3A) upon siFAM188B treatment. FOXM1 protein levels were also downregulated in the siFAM188B-treated cells (Figure 3B). MG132, a proteasome inhibitor could attenuate FOXM1 downregulation induced by siFAM188B (Figure 5B). These results are consistent with previous reports indicating that FOXM1 is regulated at the transcriptional level via positive feedback [15] and post-translational level via proteasomal degradation [19].

The regulation of ubiquitination levels is associated with the control of the proteasome degradation pathway. Several DUBs have been reported for FOXM1 regulation and their expression has been associated with cancer progression, including glioblastoma and breast cancers [9,19,20]. FOXM1 forms complexes with FAM188B (Figure 5C). FAM188B levels are positively correlated with FOXM1 levels but negatively correlated with its ubiquitination levels (Figure 3B and Figure 5D). These results indicate that FAM188B can be a regulator of FOXM1 ubiquitination. FOXM1 functions as an oncogene via transcription of genes for cell-cycle control and cell survival [6,11]. *FAM188B* knockdown induced FOXM1 downregulation along with a reduction in the proteins involved in the control of cell cycle and survival, such as CDK1, CDK2, cyclin D1, aurora kinase B, and Survivin (Figure 3C). Thus, FOXM1 downregulation might be responsible for cell growth inhibition induced by *FAM188B* knockdown.

Accumulation and nuclear translocation of β-catenin are associated with cancer development. Phosphorylation of β-catenin by GSK3β is known to induce β-catenin degradation [30]. AKT inactivates GSK3β by inducing its phosphorylation [31]. In cells with *FAM188B* knockdown, phosphorylation of GSK3β increased, and consequently, β-catenin phosphorylation decreased, which led to the accumulation of β-catenin (Figure S2). Therefore, *FAM188B* knockdown probably induces β-catenin accumulation as observed with the increase in mRNA levels (Figure 3A) and the decrease in β-catenin degradation (Figure S2), in contrast to FOXM1 downregulation. However, when de novo protein synthesis was inhibited by CHX, the level of β-catenin was diminished in a time-dependent manner (Figure 4C). This result suggests that the β-catenin level is also maintained via regulating half-life, and FAM188B could be another part of concerted regulation of β-catenin. FOXM1 is involved in translocation of β-catenin to the nucleus where it functions as a transcription factor for tumor progression [8]. FOXM1 and β-catenin were analyzed with co-immunoprecipitation assay (Figure S3). As both cytosolic and nuclear levels of FOXM1 were decreased after treatment with siFAM188B, nuclear translocation of β-catenin mediated by FOXM1 may be attenuated.

Most lung cancers are characterized based on the mutations in major oncogenes, such as *EGFR* and *K-Ras*, or tumor suppressor protein *p53* [24,32]. *FAM188B* knockdown induced cell growth inhibition regardless of these mutations as observed in A549 (*K-Ras* mutation), H1299 (*p53 null*), and PC9 (*EGFR* exon 19 deletion mutation). *FAM188B* knockdown induced FOXM1 downregulation and its regulatory proteins, including CDKs, cyclins, and Survivin (Figure 3C) in all three cell lines, indicating that FAM188B-FOXM1-CDK2/cyclins/Survivin might act as a common axis for controlling cell growth. Moreover, xenograft with siFAM188B-treated cells resulted in a retarded tumor growth (Figure 6), indicating that targeting FAM188B could be a useful anti-cancer strategy.

As for the clinical relevance of FAM188B expression in lung cancer, expressions of both FAM188B and FOXM1 are elevated in the human lung cancer tissues. The expression was minimal in the normal tissues (Figure 7A). *FAM188B* copy number increased significantly in lung adenocarcinoma and squamous cell carcinoma compared with that in adjacent normal lung tissues or blood in TCGA lung adenocarcinoma dataset (https://www.oncomine.org/resource/ui/component/dataset.html?component=d:156636501) (Figure 7B and Figure S5) and another lung cancer dataset in Oncomine (Weiss lung cancer data set; [26]) (Figure S5). Furthermore, alterations in the *FAM188B* levels were negatively correlated with overall survival (Figure 7C) as well as disease-free survival of lung cancer patients (Figure S6) in TCGA lung adenocarcinoma data set [21]. In conclusion, FAM188B expression is critical for cell growth as it regulates of FOXM1, another oncogene regulating cell cycle and survival, by its deubiquitination. Therefore, FAM188B could be a critical therapeutic target to control cancers where FAM188B or FOXM1 are overexpressed.

## Supplementary Materials

The following are available online at https://www.mdpi.com/2227-9059/8/11/465/s1, Figure S1: Effects of *FAM188B* knockdown on cell cycle (A) and cell death (B) of A549 and H1299 lung cancer cell lines at 48 h of treatment, Figure S2: Altered phospho-β-catenin and GSK3β by *FAM188B* knockdown, Figure S3: Physical interaction of FOXM1 with β-catenin in A549 and H1299 lung cancer cells, Figure S4: Expression correlation between FAM188B and FOXM1 in lung cancer tissues, Figure S5: Copy number unit variation of *FAM188B* from TCGA Lung 2 and Weiss lung statistics from Oncomine database, Figure S6: Disease-free survival significance (*p* = 0.0748) between alteration (*n* = 252) vs. non-alteration (*n* = 185) of TCGA Firehose LUAD (*n* = 586) RNAseq samples, Figure S7: Densitometric analyses of western blots of Figure 3B–D, Figure S8: Densitometric analyses of western blots of total ubiquitination of Figure 5A and co immunoprecipitation of FOXM 1 of Figure 5D, Figure S9: Original images for blots and gel electrophoresis, Table S1: Differentially expressed genes by *FAM188B* knockdown in A549 cells, Table S2: PCR primers used in this study, Table S3: Summary of immunohistochemistry analyses of FAM188B, beta-catenin, and FOXM1 with human lung tumor tissue microarray, Table S4: Immunohistochemistry score for the entire tissue microarray of lung tumors and normal lung tissues used in this study.
