# Supplementary material for: FAM188B Expression Is Critical for Cell Growth via FOXM1 Regulation in Lung Cancer
Source: Biomedicines. 2020 Oct 31;8(11):465. doi: 10.3390/biomedicines8110465 (PMC7693245; doi:10.3390/biomedicines8110465)

## Original images for Figure 1A

FAM188B GAPDH

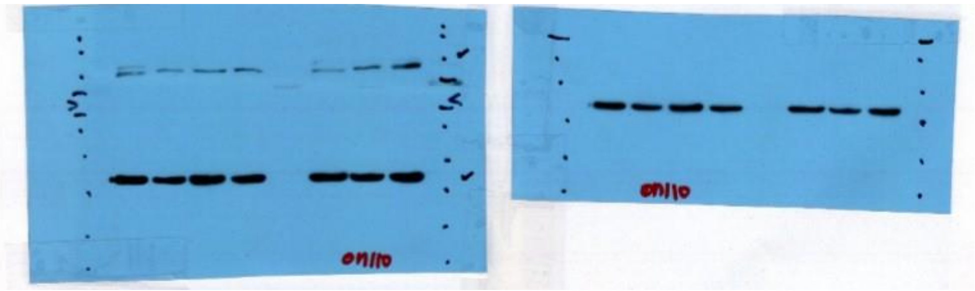

## Original images for Figure 1B

FAM188B

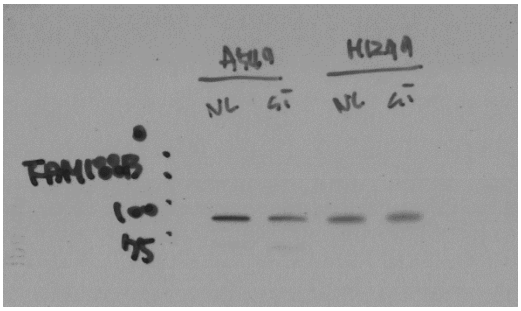


p53

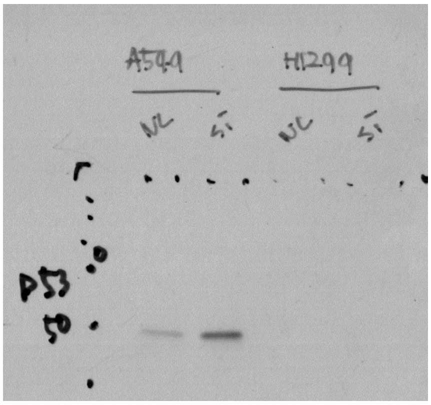


GAPDH

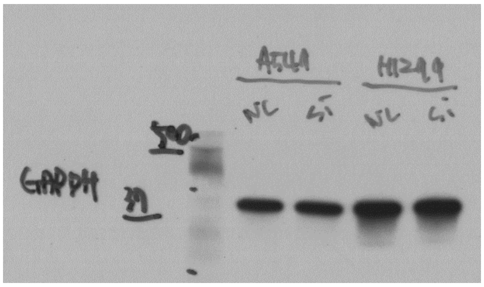


**Figure S9.** Original images of blots and gel electrophoresis

# Original images for Figure 3B

A549

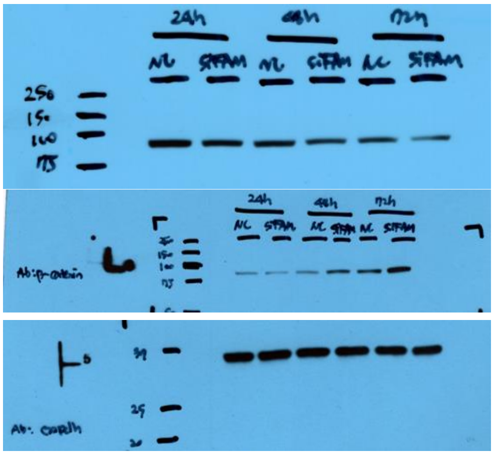


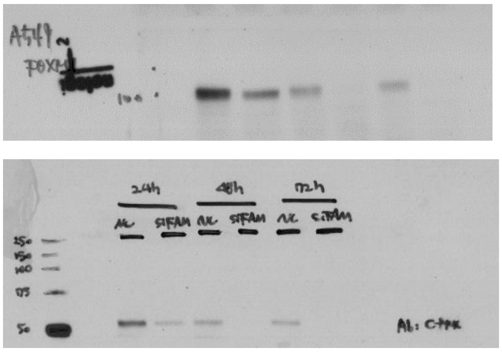


H1299

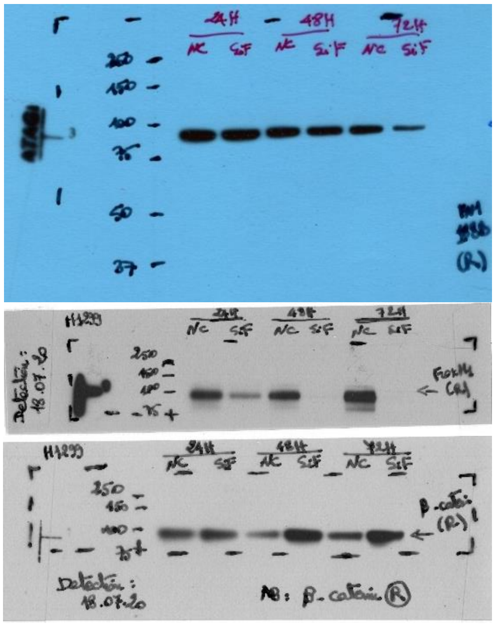


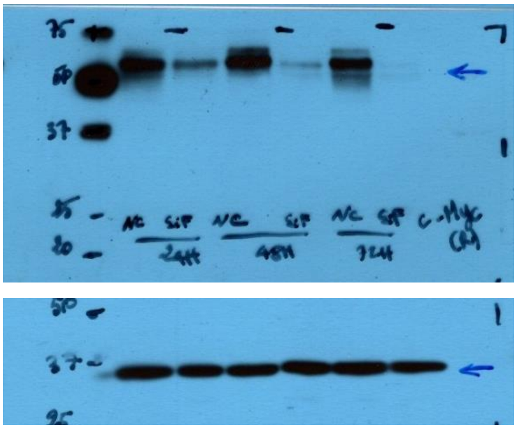


PC9

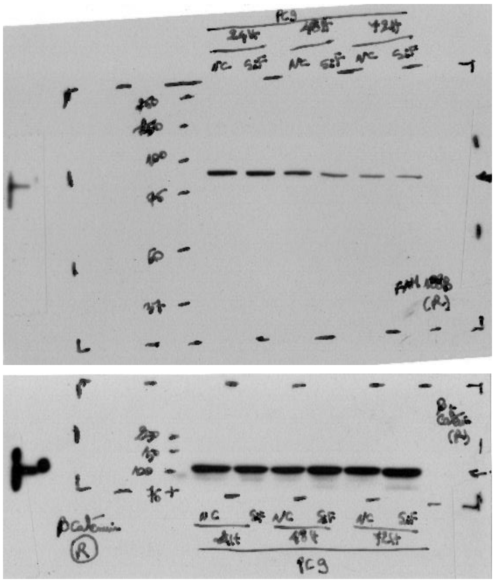


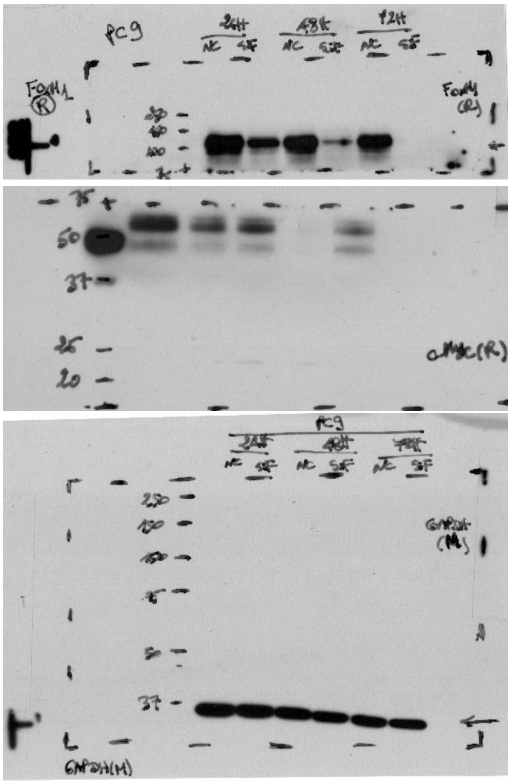

# Original images for Figure 3C

**A549**

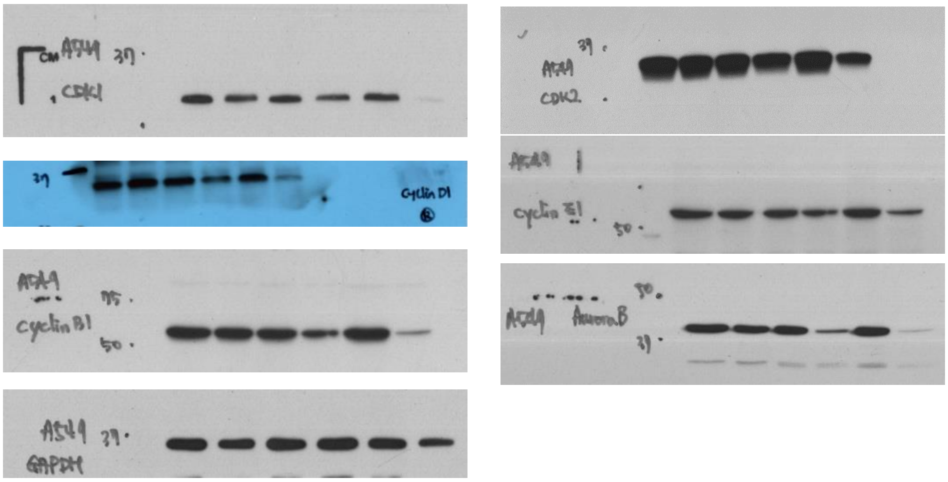


**H1299**

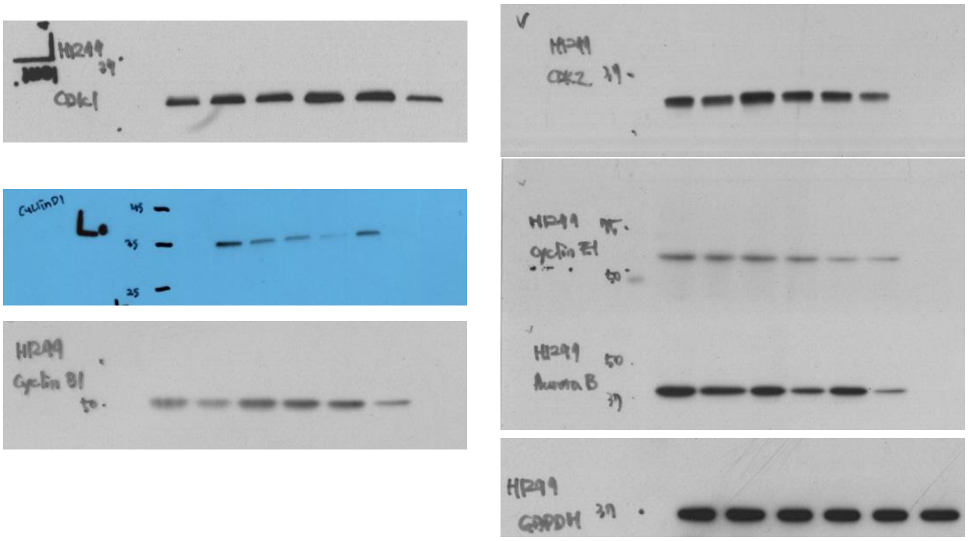


**PC9**

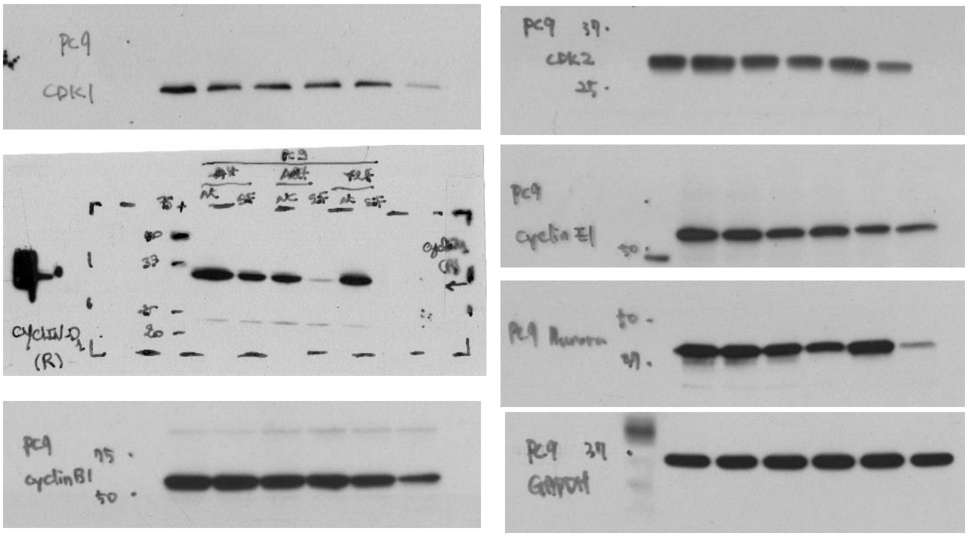

# Original images for Figure 3D

**A549**

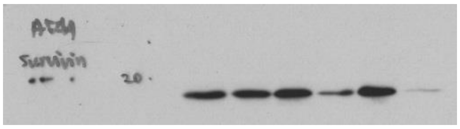


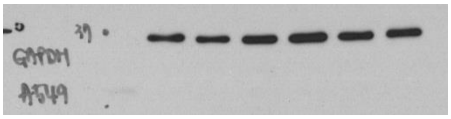


**H1299**

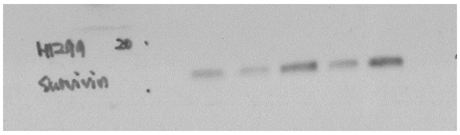


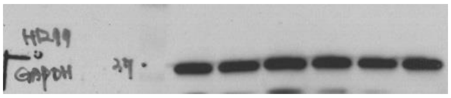


**PC9**

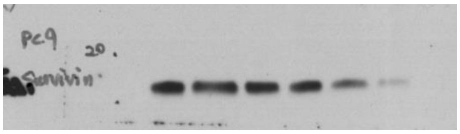


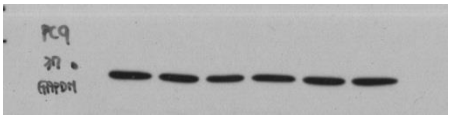


# Original images for Figure 4A

**A549**

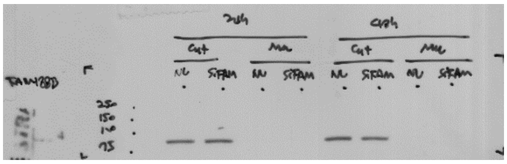

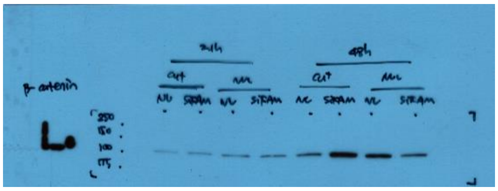

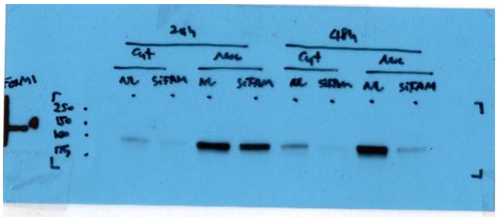

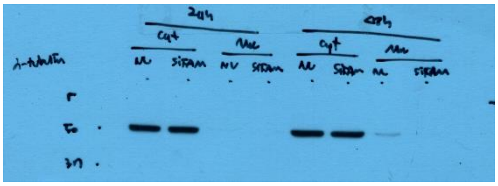

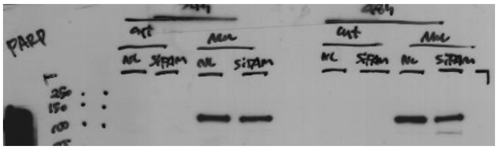


**H1299**

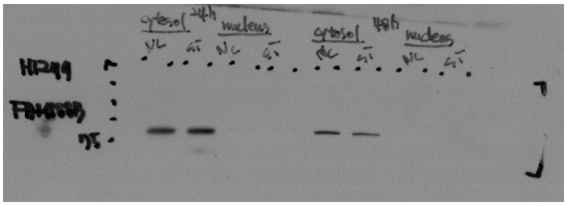


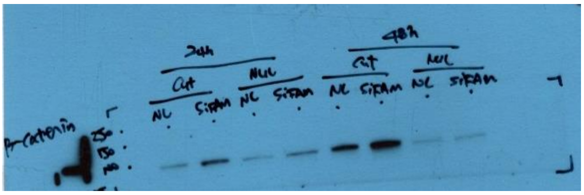

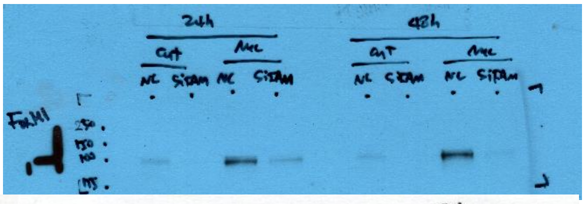

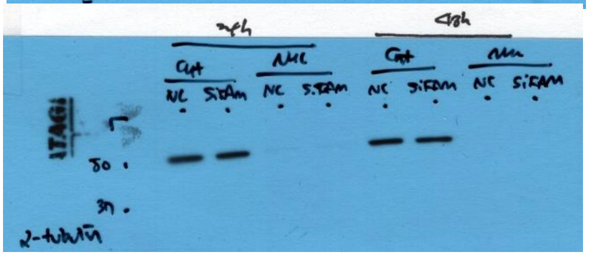

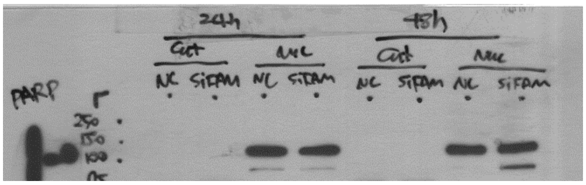

# Original images for Figure 4C

**A549**

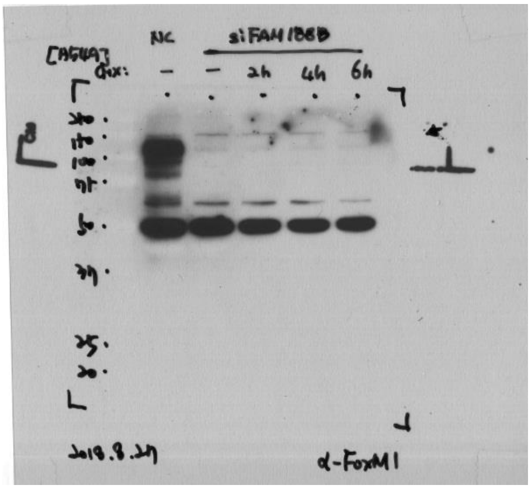


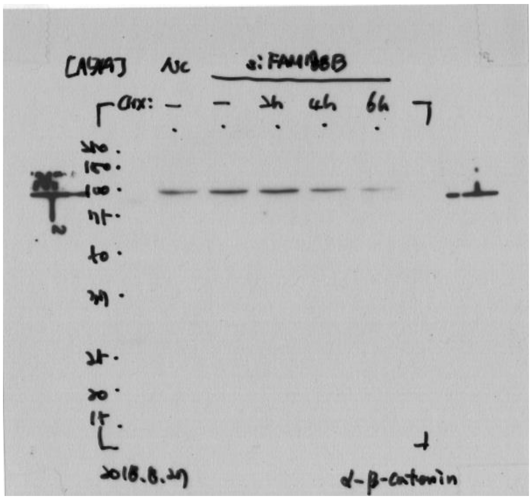


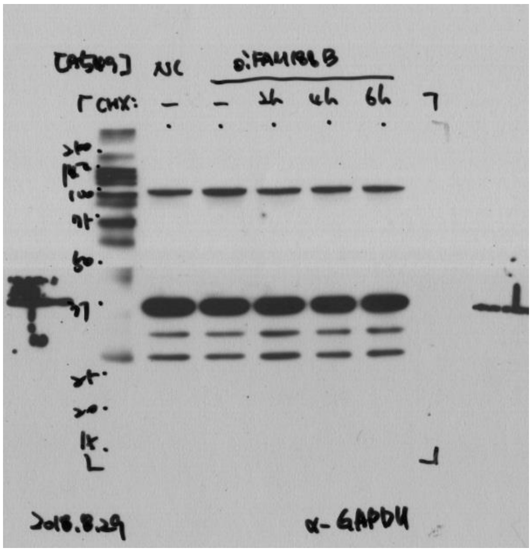


**H1299**

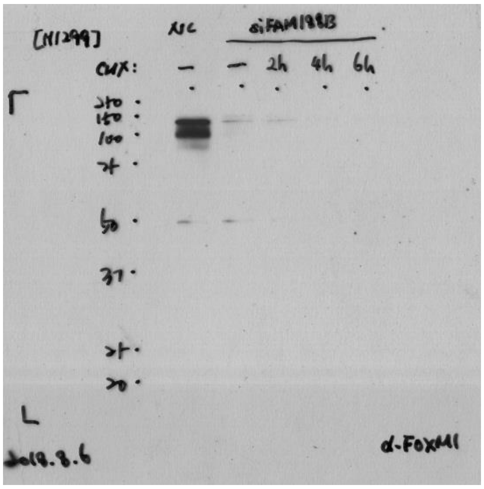


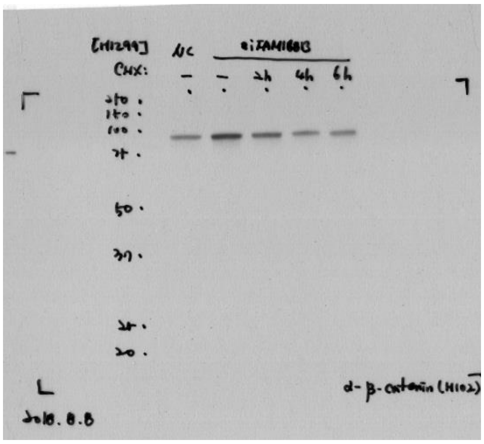


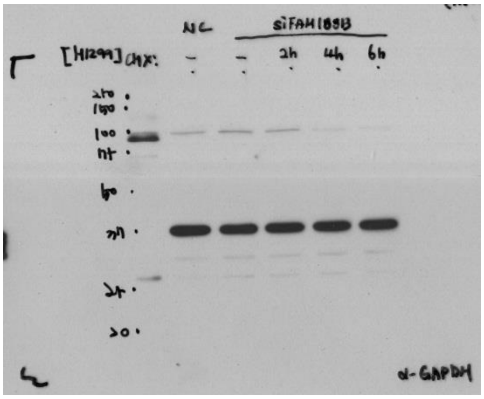

# Original images for Figure 5A

**A549**

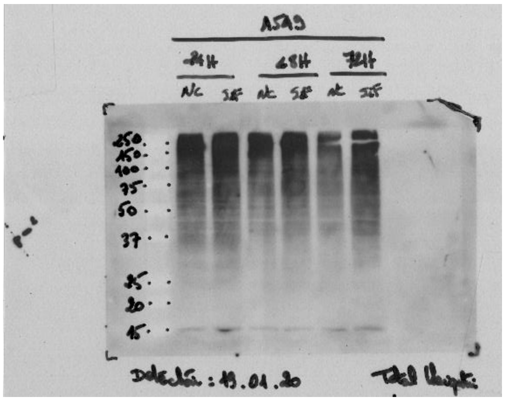


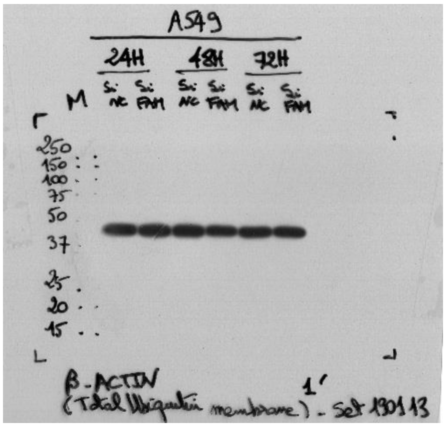


**H1299**

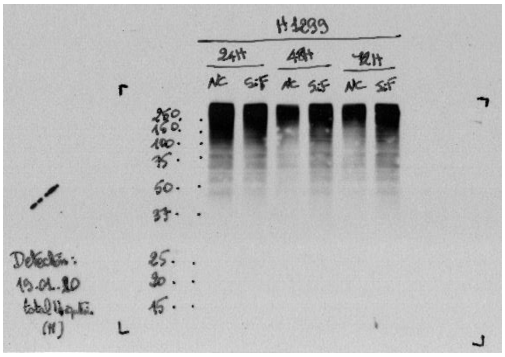


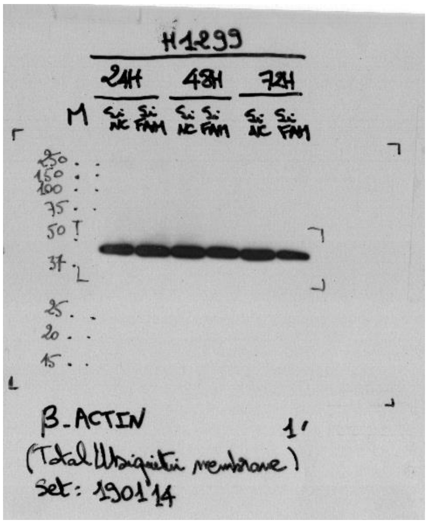


**PC9**

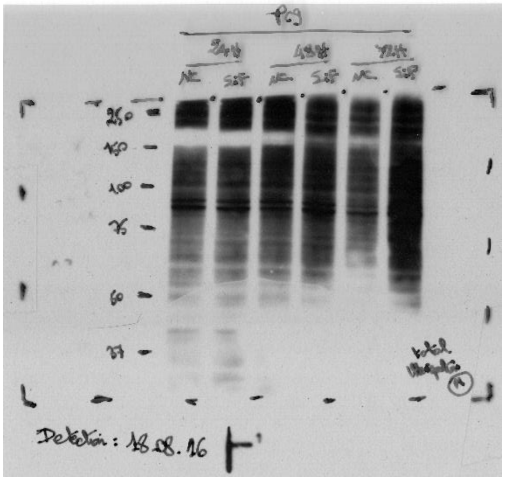


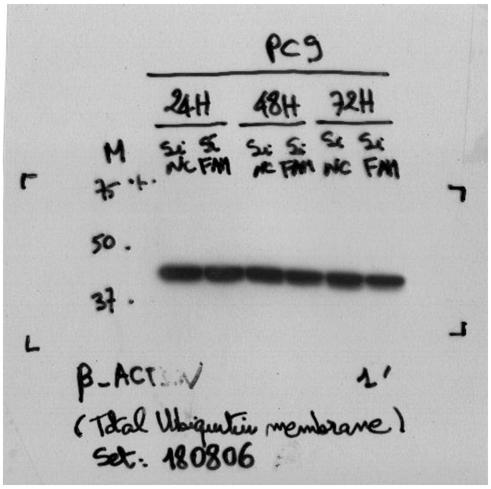

## Original images for Figure 5B

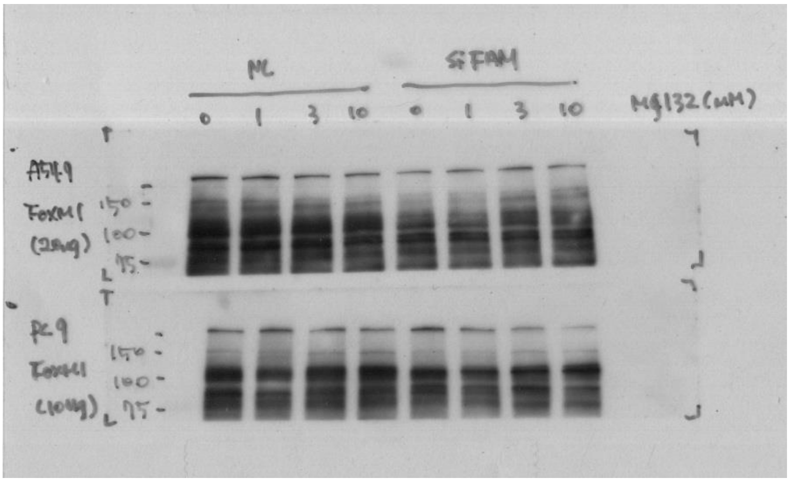


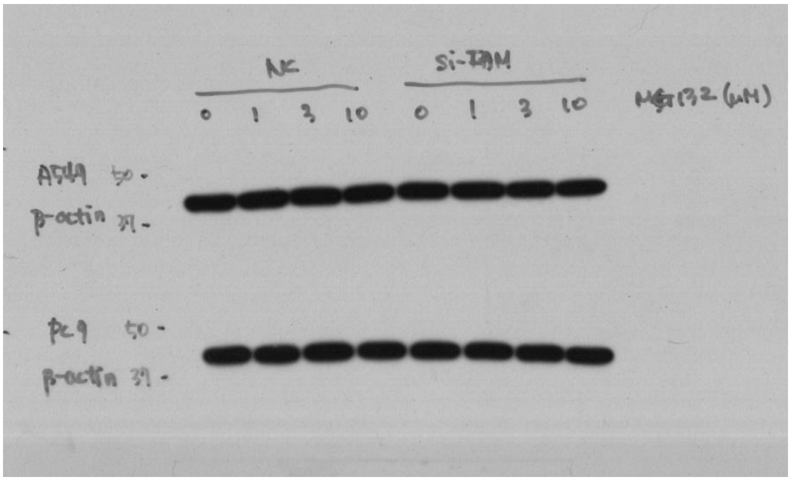


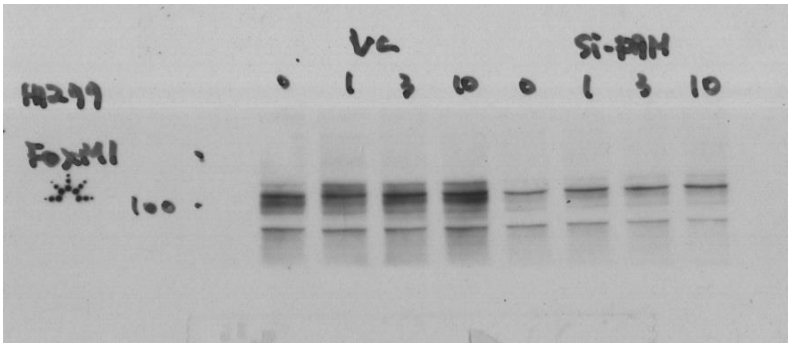


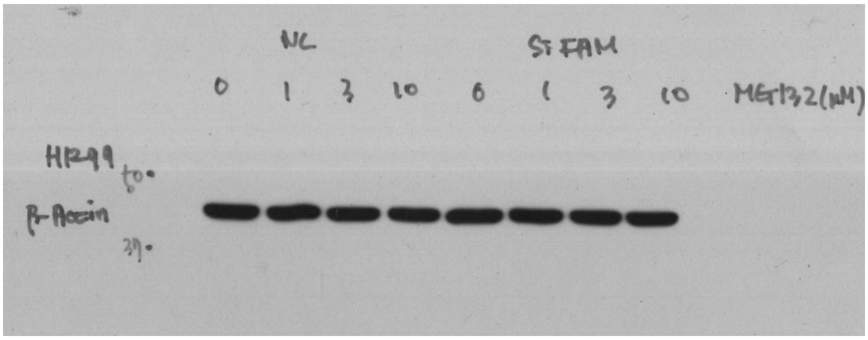

## Original images for Figure 5C

**A549**

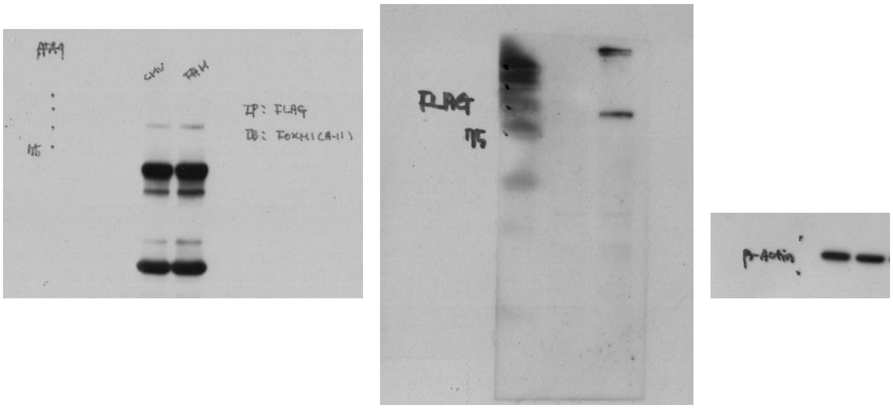


**H1299**

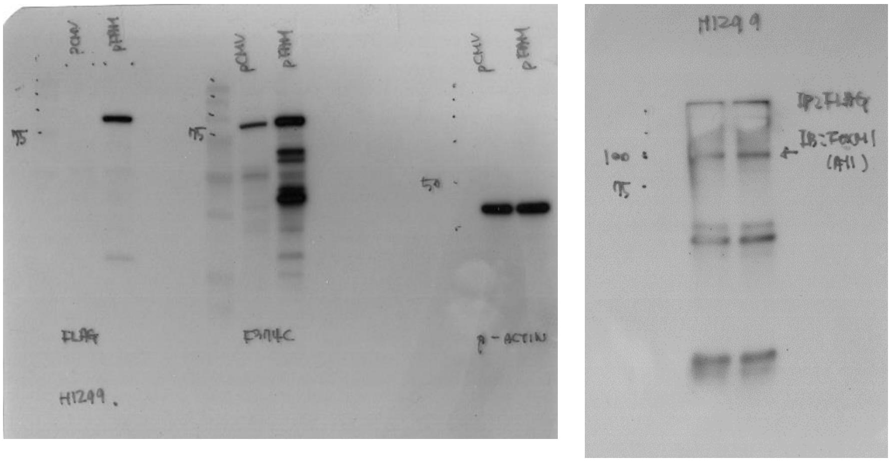


**PC9**

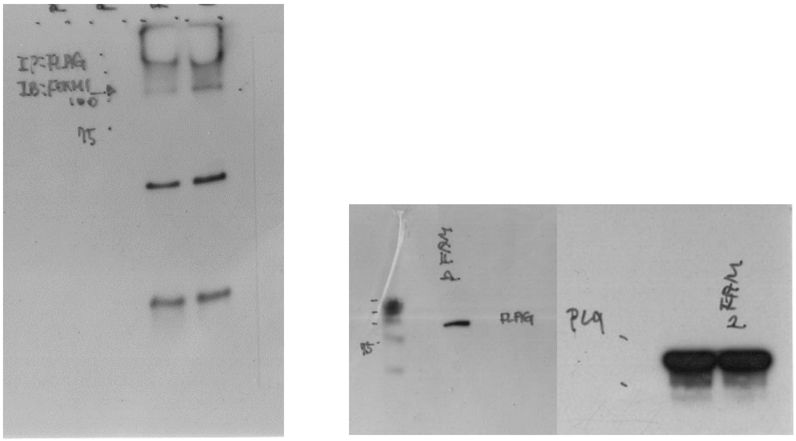

# Original images for Figure 5D

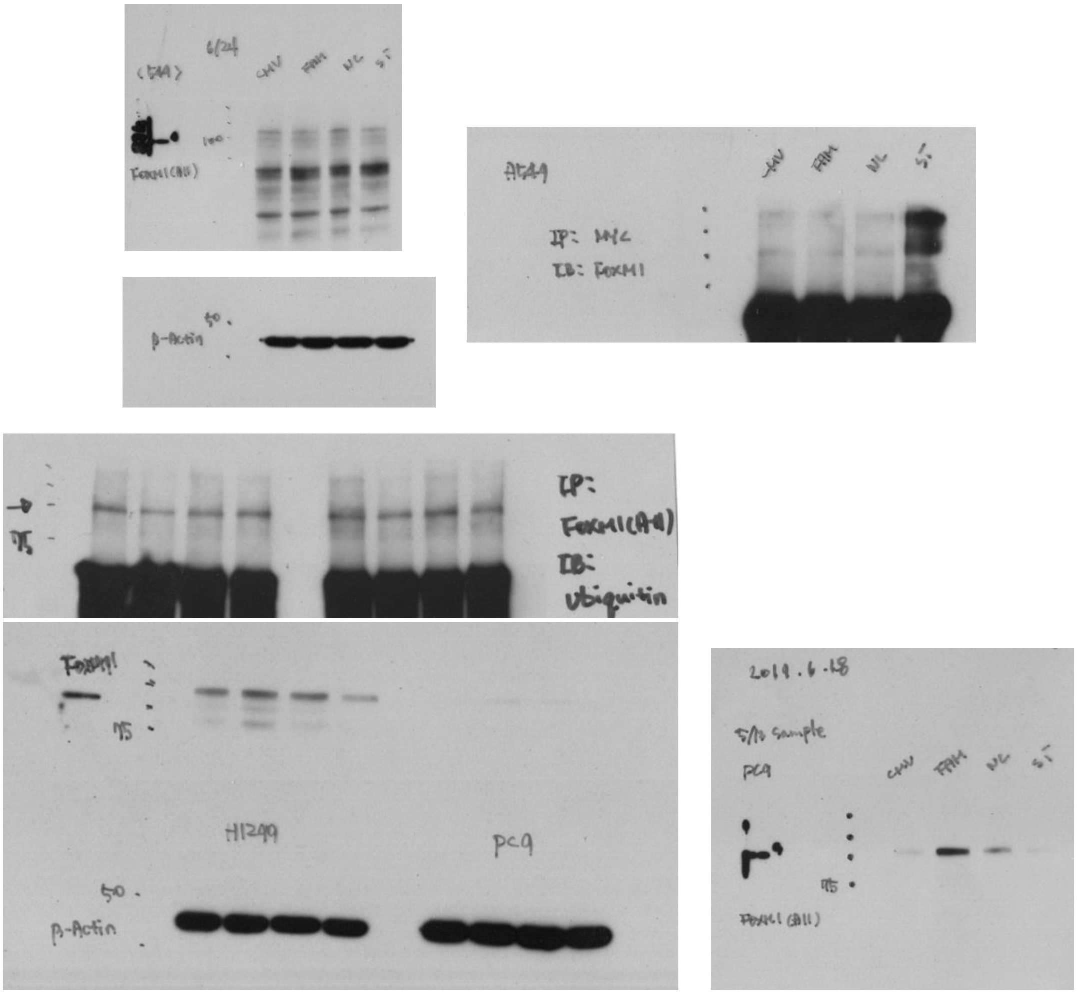


# Original images for Figure 5E

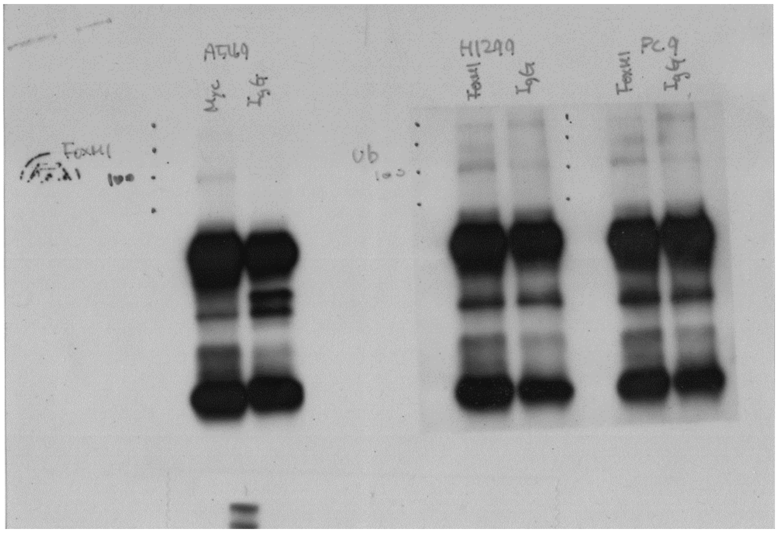

# Original images for Figure 6D

FAM188B

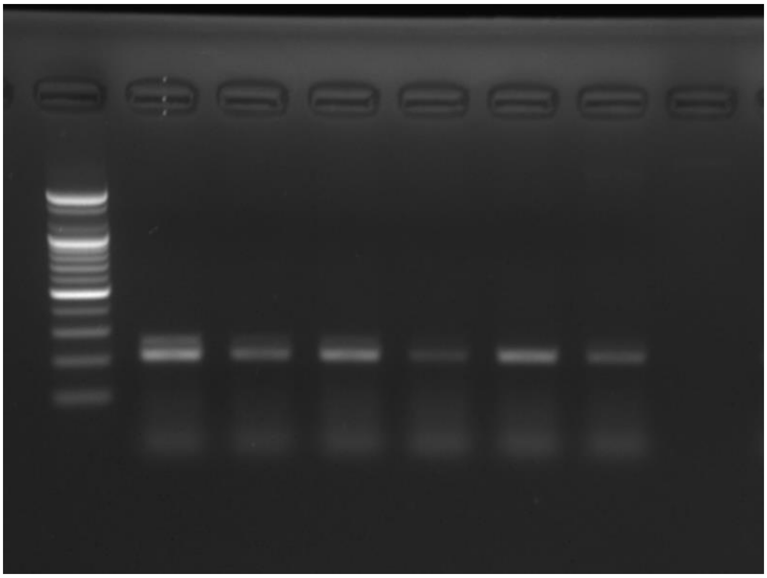

Beta-actin

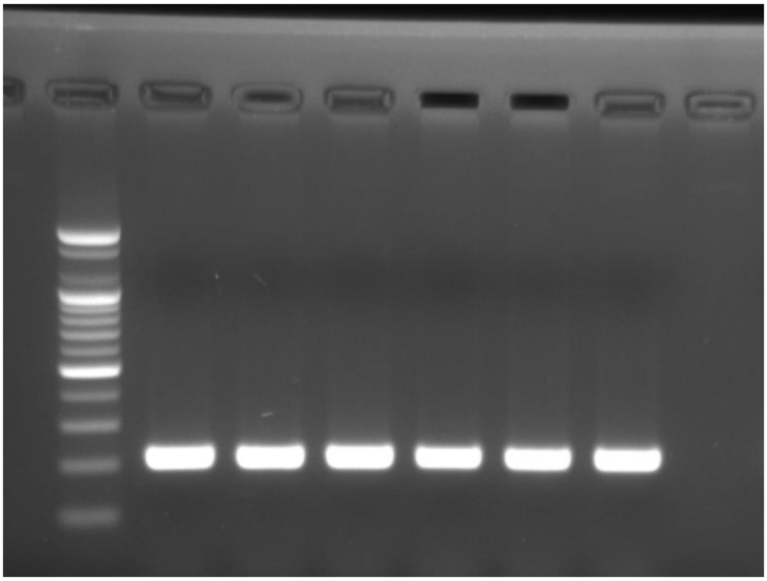

Supplement: Supplementary file 1 [file biomedicines-08-00465-s001.zip › Supplementary Figure S9-Original images of blot and gel electrophoresis.pdf]
