# Supplementary material for: FAM188B Expression Is Critical for Cell Growth via FOXM1 Regulation in Lung Cancer
Source: Biomedicines. 2020 Oct 31;8(11):465. doi: 10.3390/biomedicines8110465 (PMC7693245; doi:10.3390/biomedicines8110465)
Supplement: Supplementary file 1 [file biomedicines-08-00465-s001.zip › Supplementary Figures-S1-S8.pdf]

Supplementary Figures

FAM188B expression is critical for cell growth via FOXM1 regulation in lung cancer

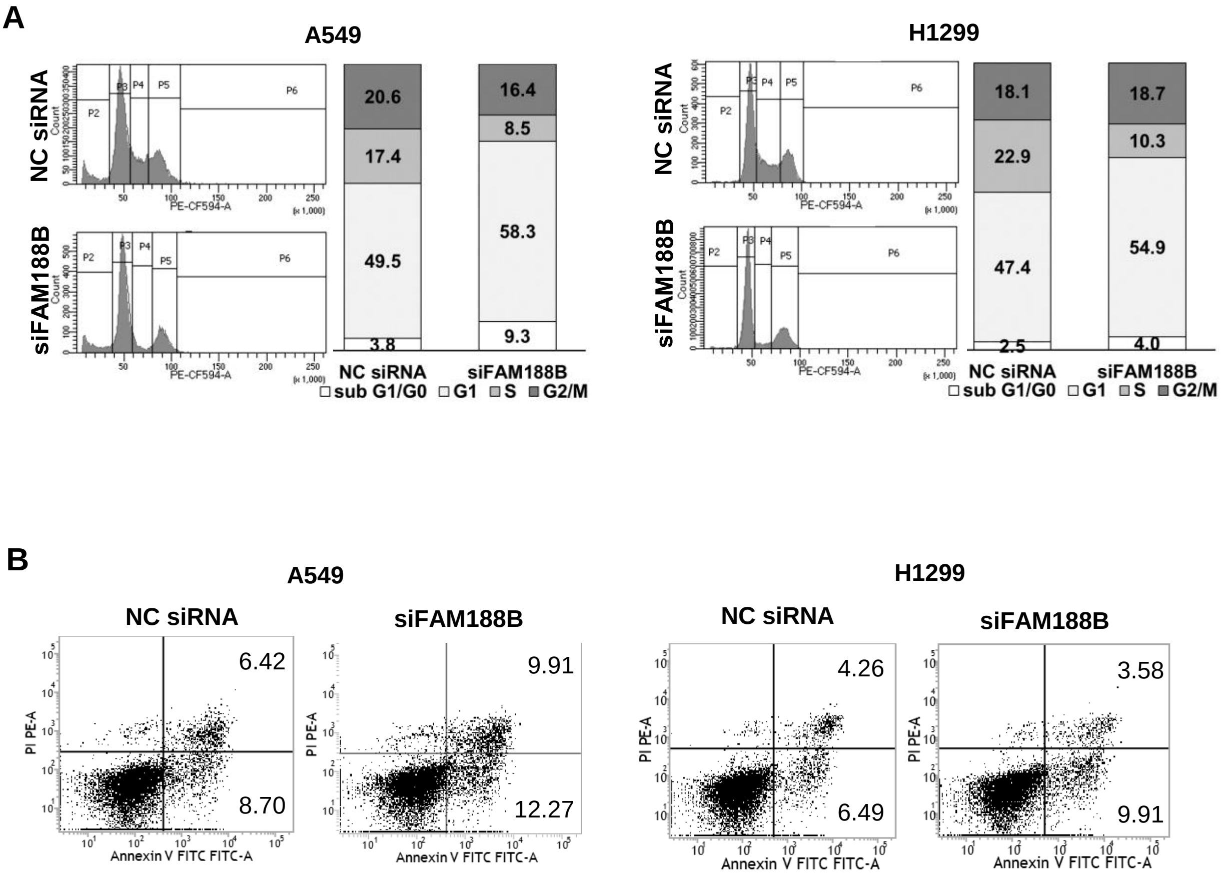

Figure S1. Effects of FAM188B knockdown on cell cycle (A) and cell death (B) of A549 and H1299 lung cancer cell lines at 48h of treatment.

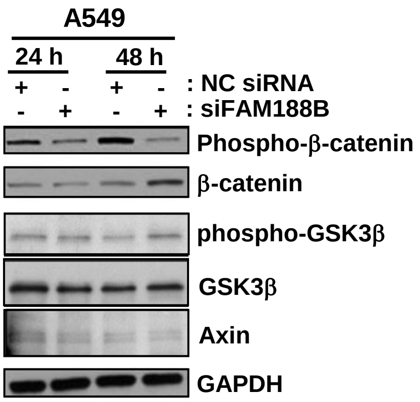

Figure S2. Altered phospho-β-catenin and GSK3β by FAM188B knockdown.

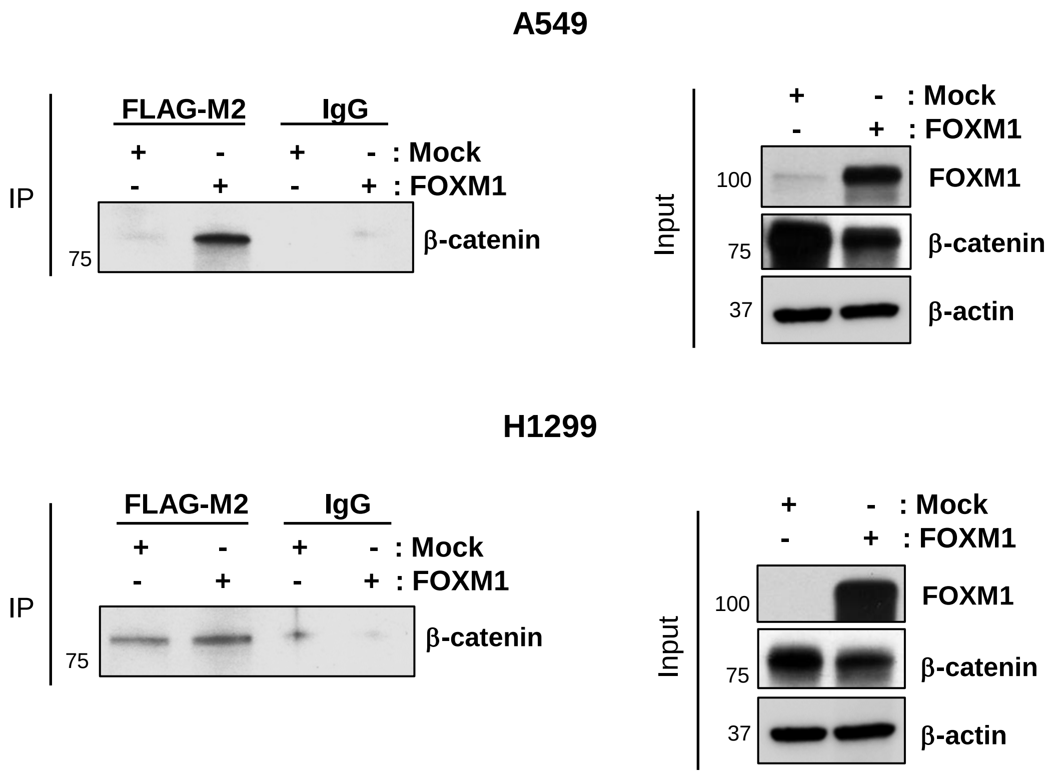

Figure S3. Physical interaction of FOXM1 with β-catenin in A549 and H1299 lung cancer cells.

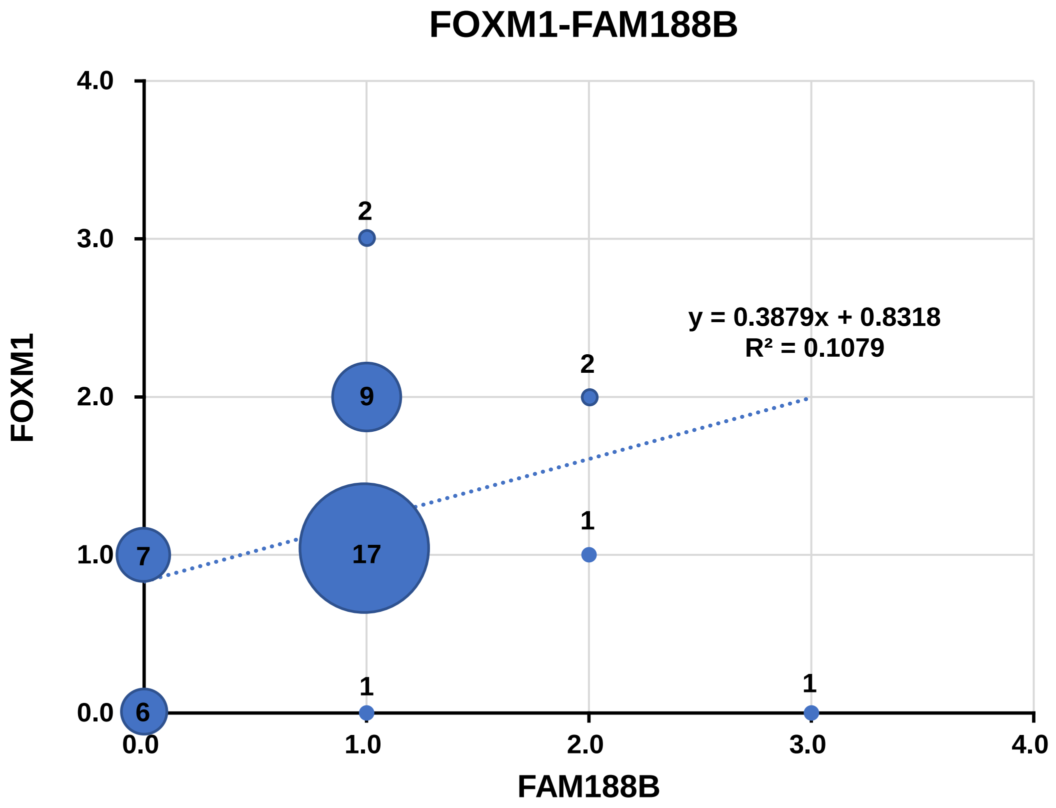

Figure S4. Expression correlation between FAM188B and FOXM1 in lung cancer tissues. Correlation coefficient was calculated by comparison of pathologic signal intensities of each immunohistochemical detection of FAM188B and FOXM1 for 46 lung cancer tissue microarray.

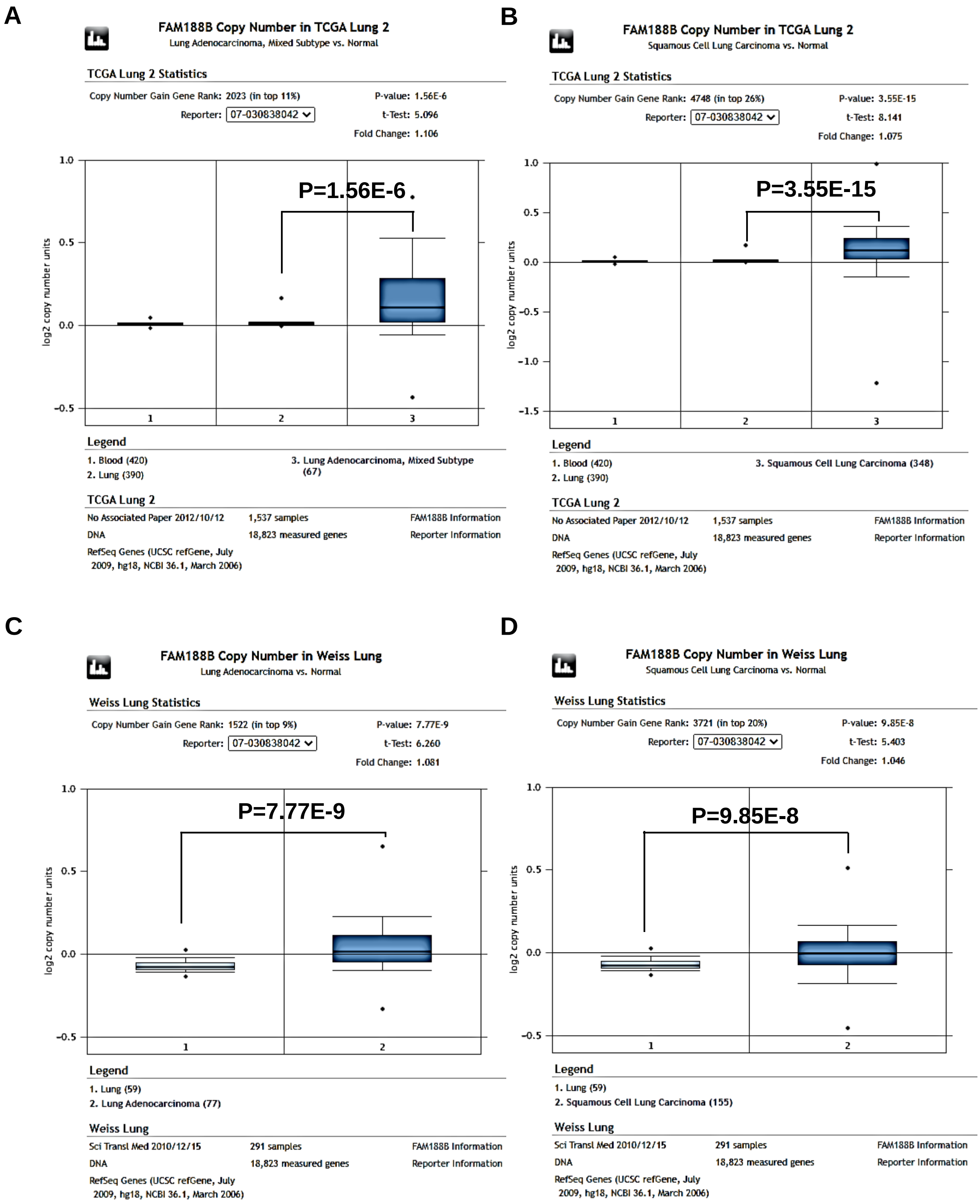

Figure S5. Copy number unit variation of FAM188B from TCGA Lung 2 and Weiss lung statistics from Oncomine database. (A) Copy number variation between normal lung tissues (*n*=390) vs. lung adenoma mixed subtypes (*n*=67) (*p*=1.56E-6). (B) Copy number variation between normal lung tissues (*n*=390) and vs. squamous cell lung carcinoma (*n*=348; *p*=3.55E-15) (C) Copy number unit variant between normal lung tissues (*n*=59) vs. lung adenoma tissues (*n*=77) (*p*=7.77E-9). (D) Copy number unit variant between normal lung tissues (*n*=59) vs. squamous cell lung carcinoma (*n*=155; *p*=9.85E-8).

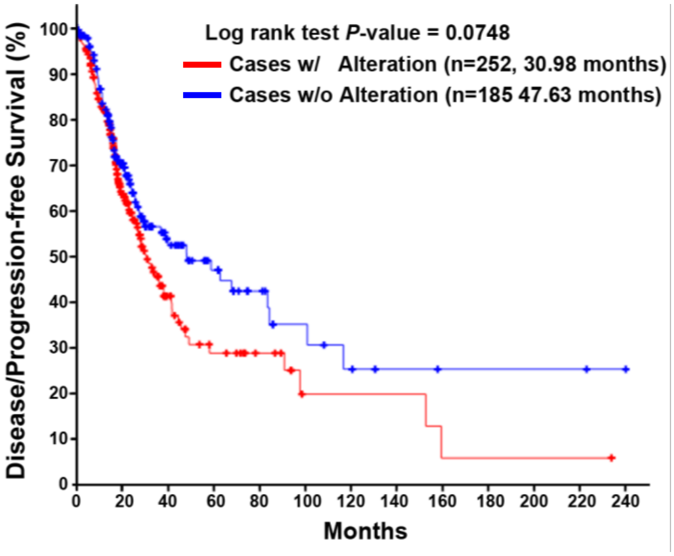

Figure S6. Disease-free survival significance (*p*=0.0748) between alteration (*n*=252) vs. non-alteration (*n*=185) of TCGA Firehose LUAD (*n*=586) RNAseq samples.

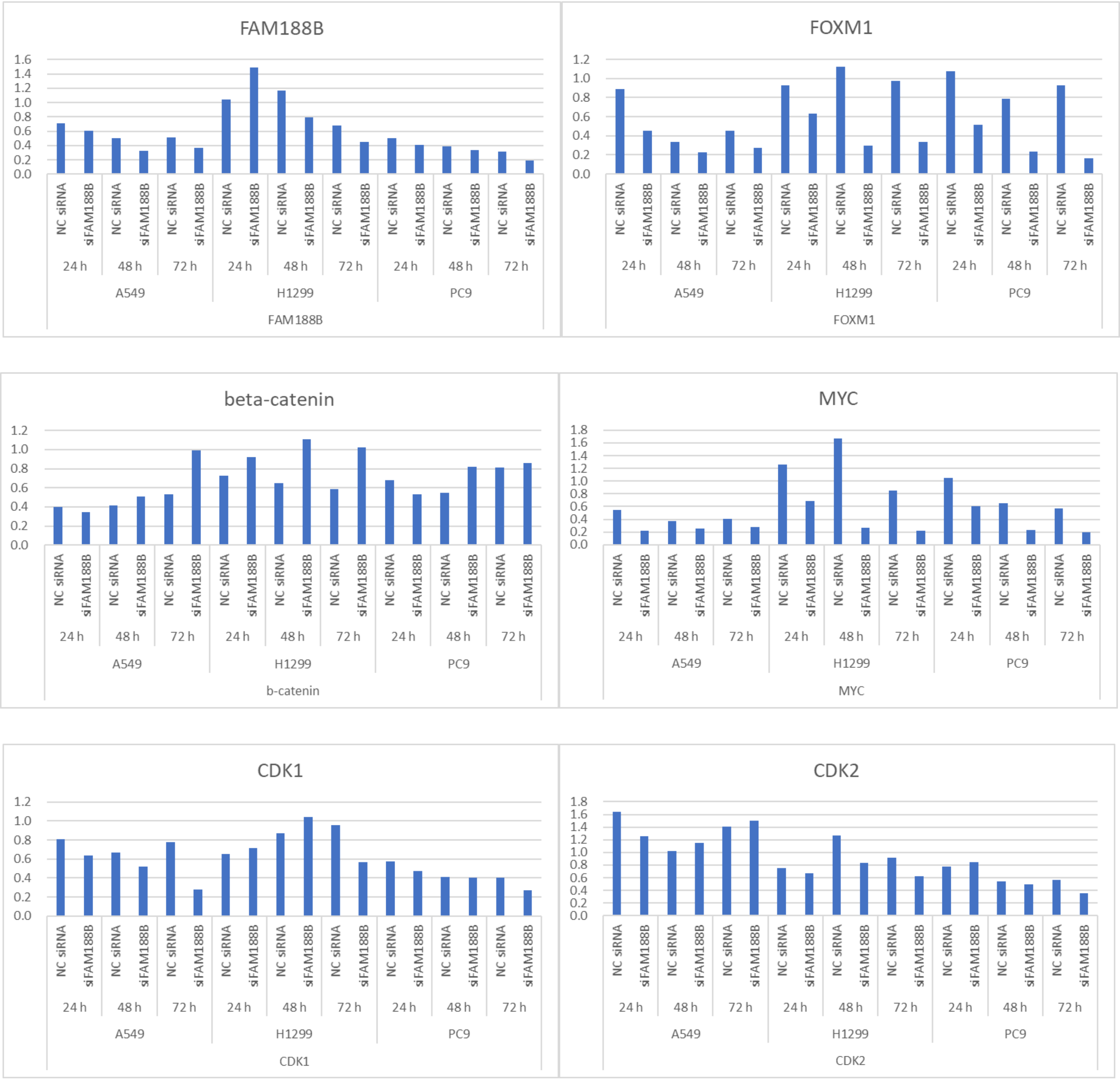

Figure S7. Densitometric analyses of western blots of Figure 3B, 3C and 3D.

(continued)

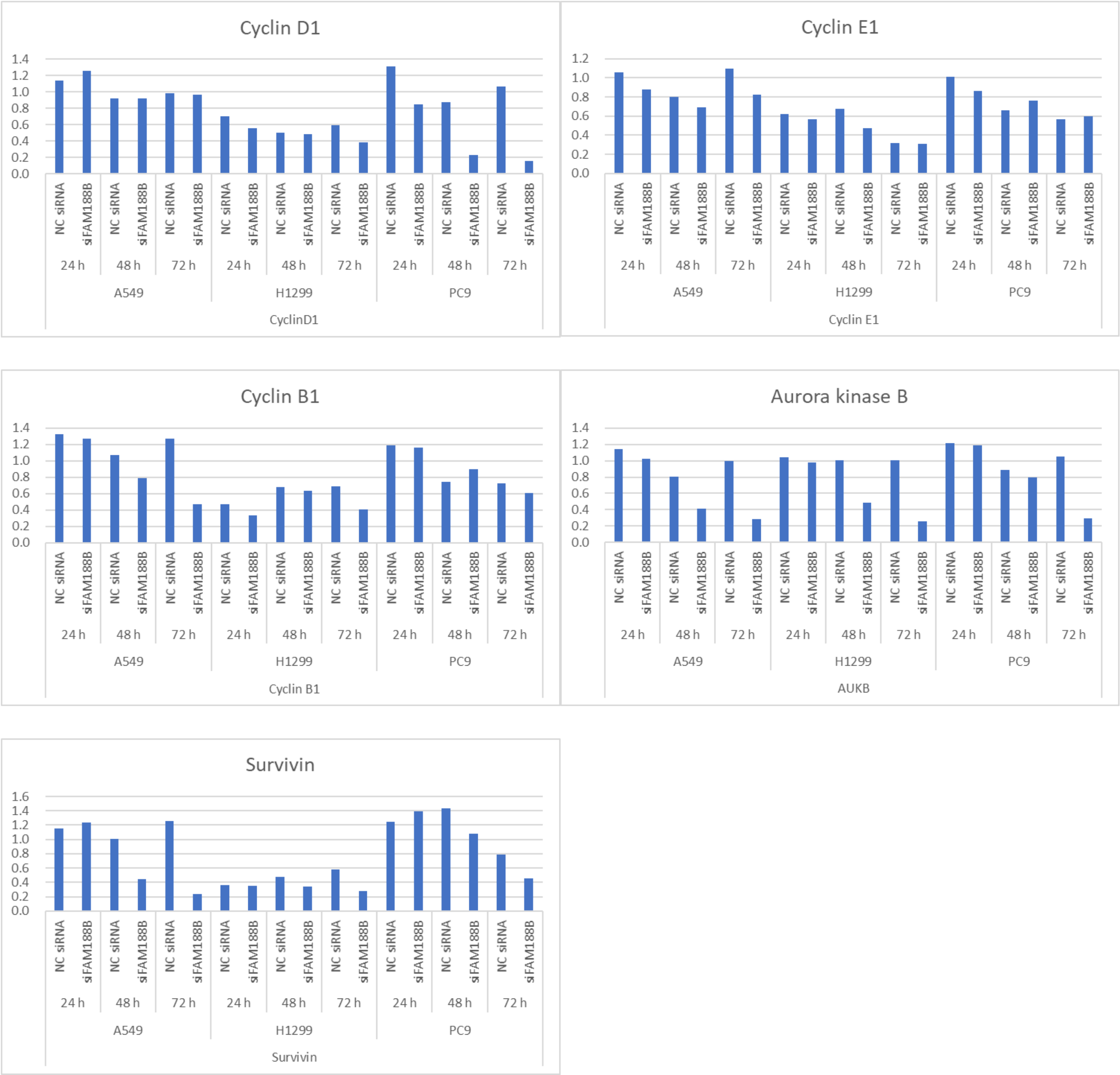

Figure S7. Densitometric analyses of western blots of Figure 3B, 3C and 3D.

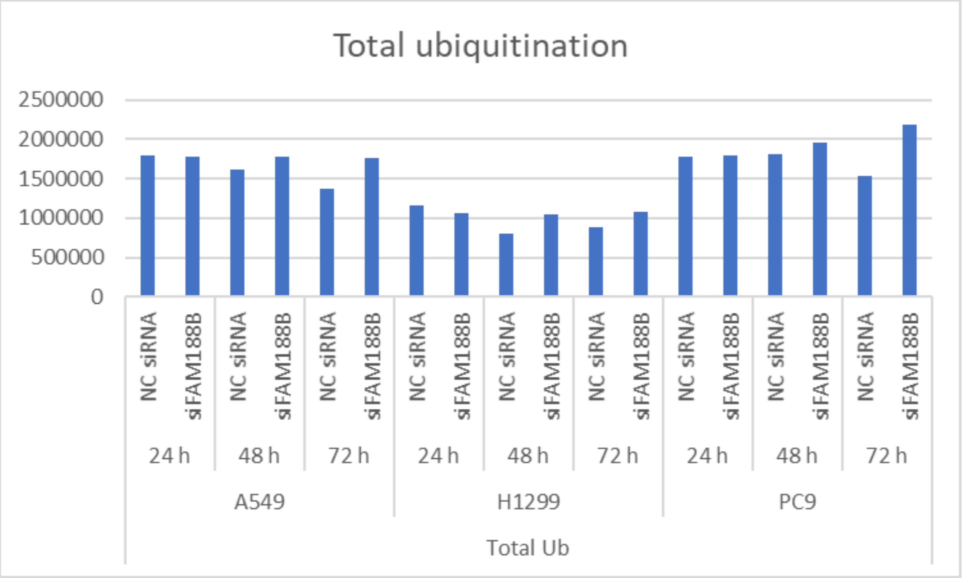

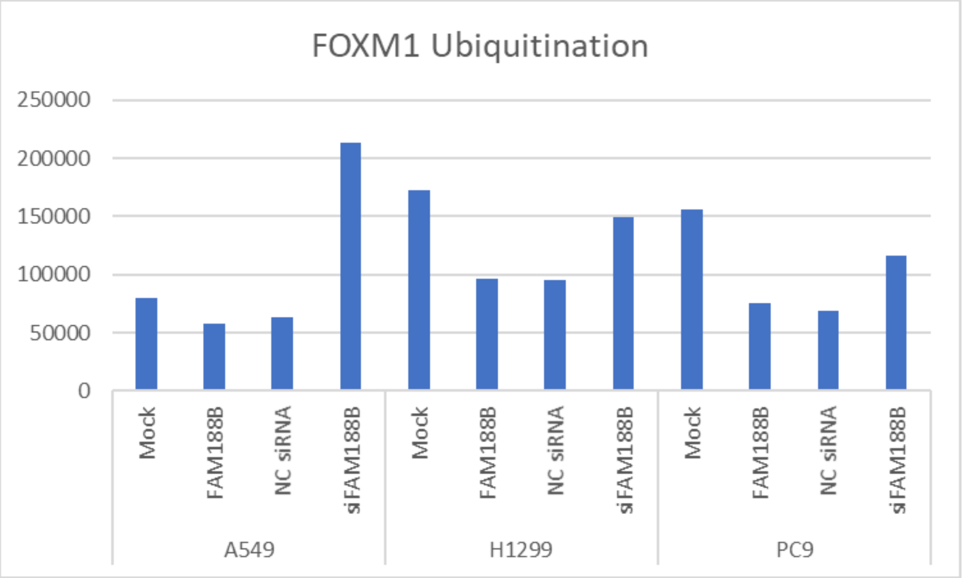

Figure S8. Densitometric analyses of western blots of total ubiquitination of Figure 5A and co-immunoprecipitation of FOXM1 of Figure 5D.
