# Supplementary material for: FAM188B Expression Is Critical for Cell Growth via FOXM1 Regulation in Lung Cancer
Source: Biomedicines. 2020 Oct 31;8(11):465. doi: 10.3390/biomedicines8110465 (PMC7693245; doi:10.3390/biomedicines8110465)
Supplement: Supplementary file 1 [file biomedicines-08-00465-s001.zip › Supplementary_Tables.pdf]

Table S1. Differentially expressed genes by FAM188B knockdown in A549 cells.

ID	logFC	P.Value	ID2	logFC3	P.Value4	ID	logFC	P.Value	ID	logFC	P.Value
L3MBTL3	1.75	3.00E-04	CENPF	-2.33	1.00E-04	ABCA1	1.87	5.00E-05	TNKS	1.58	5.00E-05
ERO1LB	1.58	3.00E-04	CORO1A	-2.64	1.00E-04	LIPH	1.87	5.00E-05	CXXC5	1.57	5.00E-05
SGPP2	-1.59	3.00E-04	ARTN	-2.85	1.00E-04	EID1	1.86	5.00E-05	FYTTD1	1.57	5.00E-05
CT60	-1.9	3.00E-04	LINC00473	-2.9	1.00E-04	KLHL15	1.86	5.00E-05	GSPT2	1.57	5.00E-05
SYNE2	-1.9	3.00E-04	ZNF418	-3.5	1.00E-04	TDP2	1.86	5.00E-05	NUFIP2	1.57	5.00E-05
IL1R2	-2.11	3.00E-04	MAP2	4.03	5.00E-05	ASF1A	1.84	5.00E-05	PDGFC	1.57	5.00E-05
ZNF519	-2.85	3.00E-04	ACER2	3.41	5.00E-05	CHIC1	1.84	5.00E-05	SDCBP	1.57	5.00E-05
SLC26A9	-2.91	3.00E-04	CTSO	3.31	5.00E-05	PLEKHF2	1.84	5.00E-05	MTMR9	1.56	5.00E-05
HFM1	2.99	0.00025	SLC7A11	3.25	5.00E-05	YPEL5	1.84	5.00E-05	PREPL	1.56	5.00E-05
CPEB1	1.98	0.00025	ZMAT3	3.14	5.00E-05	FRS2	1.83	5.00E-05	TIPARP	1.56	5.00E-05
CYP1A1	1.96	0.00025	SLCO2B1	3.13	5.00E-05	LIN7C	1.83	5.00E-05	FBXW7	1.55	5.00E-05
TNFRSF9	1.75	0.00025	DSC2	3.1	5.00E-05	SKIL	1.83	5.00E-05	KDM5B	1.55	5.00E-05
APLF	1.72	0.00025	ZBTB41	3.09	5.00E-05	ZNF678	1.83	5.00E-05	MBTPS2	1.55	5.00E-05
FRAT1	1.67	0.00025	GXYLT1	2.78	5.00E-05	GMFB	1.82	5.00E-05	PRKAB2	1.55	5.00E-05
FYN	1.54	0.00025	HIST1H2BC	2.77	5.00E-05	NFE2L2	1.82	5.00E-05	TBL1XR1	1.55	5.00E-05
NPY4R	-1.55	0.00025	KRTAP4-1	2.77	5.00E-05	PGAP1	1.82	5.00E-05	ATF3	1.54	5.00E-05
CCDC120	-1.58	0.00025	LY9	2.76	5.00E-05	DNAJC27	1.81	5.00E-05	CCNC	1.54	5.00E-05
MUC5AC	-1.69	0.00025	RRM2B	2.74	5.00E-05	DSG2	1.81	5.00E-05	CCNG1	1.54	5.00E-05
RAB26	-1.78	0.00025	DTNA	2.71	5.00E-05	HDAC9	1.81	5.00E-05	FAM199X	1.54	5.00E-05
NUPR1	-1.94	0.00025	SESN1	2.67	5.00E-05	RGS17	1.81	5.00E-05	IREB2	1.54	5.00E-05
IGFN1	-2.1	0.00025	SLFN5	2.63	5.00E-05	SPOPL	1.81	5.00E-05	RFK	1.54	5.00E-05
RNF212	-2.22	0.00025	CPEB3	2.57	5.00E-05	FBXO30	1.8	5.00E-05	ZBTB10	1.54	5.00E-05
ABHD16B	-2.27	0.00025	F2RL2	2.56	5.00E-05	PLEKHA1	1.8	5.00E-05	DRAM1	1.53	5.00E-05
AC130352.1	-2.32	0.00025	UHMK1	2.47	5.00E-05	PPM1D	1.8	5.00E-05	GADD45A	1.53	5.00E-05
CKMT1B	-2.39	0.00025	SAMD5	2.45	5.00E-05	SLC40A1	1.8	5.00E-05	ROR1	1.53	5.00E-05
MAFF	1.51	2.00E-04	VPS4B	2.45	5.00E-05	MAP3K1	1.79	5.00E-05	TAF13	1.53	5.00E-05
IGSF11	-1.57	2.00E-04	BTG2	2.44	5.00E-05	KLHL4	1.78	5.00E-05	TDRD7	1.53	5.00E-05
CBX2	-1.6	2.00E-04	KPNA5	2.43	5.00E-05	POF1B	1.78	5.00E-05	TMEM128	1.53	5.00E-05
ATN1	-1.61	2.00E-04	OTUD1	2.41	5.00E-05	SEPSECS	1.78	5.00E-05	DNAJB14	1.52	5.00E-05
RTN4R	-1.69	2.00E-04	ABCA12	2.4	5.00E-05	GNA13	1.77	5.00E-05	GTF2A1	1.52	5.00E-05
AGMO	-1.82	2.00E-04	UBL3	2.39	5.00E-05	SH3BGRL	1.77	5.00E-05	GTF3C3	1.52	5.00E-05
WNK2	-1.91	2.00E-04	ARRDC3	2.37	5.00E-05	SRPK2	1.77	5.00E-05	PTPRJ	1.52	5.00E-05
JMJD8	-1.93	2.00E-04	CREBRF	2.37	5.00E-05	CYLD	1.76	5.00E-05	RNASEL	1.52	5.00E-05
MAP2K6	-1.94	2.00E-04	FAM73A	2.37	5.00E-05	PPP1R14C	1.76	5.00E-05	TLE4	1.52	5.00E-05
BLM	-2.04	2.00E-04	IL6ST	2.36	5.00E-05	STX6	1.76	5.00E-05	PGGT1B	1.51	5.00E-05
SLC2A14	-2.04	2.00E-04	ZDBF2	2.35	5.00E-05	AKR1C1	1.75	5.00E-05	RAP2C	1.51	5.00E-05
LCN12	-2.17	2.00E-04	LRP12	2.33	5.00E-05	CLIP4	1.75	5.00E-05	CCDC138	-1.51	5.00E-05
WNT10B	-2.47	2.00E-04	BACH1	2.32	5.00E-05	PLOD2	1.75	5.00E-05	CENPE	-1.51	5.00E-05
IFI44L	-2.92	2.00E-04	ADAM9	2.31	5.00E-05	ZBTB6	1.75	5.00E-05	HSD17B8	-1.51	5.00E-05
SLC51B	-2.94	2.00E-04	EPB41L5	2.31	5.00E-05	SUCO	1.74	5.00E-05	SUOX	-1.51	5.00E-05
CD74	-3.6	2.00E-04	CA8	2.29	5.00E-05	TNFRSF10D	1.74	5.00E-05	APBA2	-1.52	5.00E-05
BACH2	3.48	0.00015	ZDHHC20	2.29	5.00E-05	AKIRIN1	1.73	5.00E-05	FERMT1	-1.52	5.00E-05
CLDN16	2.96	0.00015	SPATA18	2.28	5.00E-05	C12orf5	1.73	5.00E-05	H2AFX	-1.52	5.00E-05
FAM78A	2.16	0.00015	ARHGAP5	2.27	5.00E-05	GBE1	1.73	5.00E-05	SLC10A3	-1.52	5.00E-05
CYS1	2.12	0.00015	CSGALNACT2	2.27	5.00E-05	ATP11B	1.72	5.00E-05	SNAPIN	-1.52	5.00E-05
FBXL17	1.81	0.00015	RARB	2.27	5.00E-05	C7orf73	1.72	5.00E-05	B4GALT2	-1.53	5.00E-05
YIPF4	1.79	0.00015	BMF	2.26	5.00E-05	KLHL5	1.72	5.00E-05	BASP1	-1.53	5.00E-05
PAQR8	1.77	0.00015	LCOR	2.26	5.00E-05	XRN1	1.72	5.00E-05	CCDC34	-1.53	5.00E-05
IL1R1	1.76	0.00015	IPMK	2.24	5.00E-05	ADAM23	1.71	5.00E-05	EPB49	-1.53	5.00E-05
ZFP37	1.76	0.00015	HBP1	2.23	5.00E-05	CDC37L1	1.71	5.00E-05	TMED9	-1.53	5.00E-05
FAM160B1	1.75	0.00015	MICU3	2.23	5.00E-05	NAA30	1.71	5.00E-05	ALKBH2	-1.54	5.00E-05
SYDE2	1.75	0.00015	PTGR1	2.21	5.00E-05	NEFL	1.71	5.00E-05	DDIT4	-1.54	5.00E-05
LRIF1	1.58	0.00015	SLC39A10	2.21	5.00E-05	RB1CC1	1.71	5.00E-05	EZH2	-1.54	5.00E-05
PCMTD1	1.55	0.00015	LOXL4	2.2	5.00E-05	RIT1	1.71	5.00E-05	FOXQ1	-1.54	5.00E-05
PPARD	-1.59	0.00015	ANKRD46	2.19	5.00E-05	BLOC1S2	1.7	5.00E-05	KIF14	-1.54	5.00E-05
AHNAK2	-1.64	0.00015	CYP4F11	2.17	5.00E-05	C7orf60	1.7	5.00E-05	MPI	-1.54	5.00E-05
TEKT4P2	-1.7	0.00015	FAM63B	2.17	5.00E-05	GATA6	1.7	5.00E-05	SLC7A5	-1.54	5.00E-05
VDR	-1.74	0.00015	FCHO2	2.14	5.00E-05	YPEL2	1.7	5.00E-05	TNK2	-1.54	5.00E-05
ABCB9	-1.75	0.00015	CYP4F3	2.13	5.00E-05	MOB1A	1.69	5.00E-05	UBE2T	-1.54	5.00E-05
SLC39A3	-1.78	0.00015	LIN7A	2.13	5.00E-05	PRKAA2	1.69	5.00E-05	UBL7	-1.54	5.00E-05
EIF4G1	-1.83	0.00015	MTX3	2.13	5.00E-05	SBNO1	1.69	5.00E-05	CCDC124	-1.55	5.00E-05
RTN4RL2	-1.9	0.00015	SLC30A1	2.12	5.00E-05	SORBS1	1.69	5.00E-05	DNA2	-1.55	5.00E-05
CALHM2	-2	0.00015	JMY	2.11	5.00E-05	TNKS2	1.69	5.00E-05	LMNB1	-1.55	5.00E-05
TTF2	-2	0.00015	HPD	2.1	5.00E-05	ZKSCAN2	1.69	5.00E-05	LTBP4	-1.55	5.00E-05
KNTC1	-2.42	0.00015	GPC6	2.08	5.00E-05	ERMP1	1.68	5.00E-05	TGFB1I1	-1.55	5.00E-05
RIBC2	-3.85	0.00015	JHDM1D	2.07	5.00E-05	MIER3	1.67	5.00E-05	TMEM201	-1.55	5.00E-05
ZNF425	2.41	1.00E-04	RP11-363E7.4	2.07	5.00E-05	PJA2	1.67	5.00E-05	EBP	-1.56	5.00E-05
PDE2A	2	1.00E-04	TNPO1	2.07	5.00E-05	SPRED1	1.67	5.00E-05	ITGB5	-1.56	5.00E-05
MOB1B	1.75	1.00E-04	CCDC71L	2.06	5.00E-05	ACAP2	1.66	5.00E-05	MEN1	-1.56	5.00E-05
BNIP3P1	1.67	1.00E-04	MIB1	2.05	5.00E-05	APOL6	1.66	5.00E-05	RBM42	-1.56	5.00E-05
PLEKHM3	1.63	1.00E-04	HIST2H2BE	2.04	5.00E-05	ATP6V1A	1.66	5.00E-05	RRM1	-1.56	5.00E-05
MAN1A2	1.61	1.00E-04	ADAM17	2.02	5.00E-05	EFR3A	1.66	5.00E-05	SLC35F6	-1.56	5.00E-05
ARNTL2	1.54	1.00E-04	CNRIP1	2	5.00E-05	ZNF24	1.66	5.00E-05	UCP2	-1.56	5.00E-05
OSGIN2	1.54	1.00E-04	GLCE	2	5.00E-05	PLCL2	1.65	5.00E-05	C16orf74	-1.57	5.00E-05
MYADM	-1.54	1.00E-04	UNC119B	1.99	5.00E-05	RRAS2	1.65	5.00E-05	MYO1C	-1.57	5.00E-05
TFAP4	-1.54	1.00E-04	EBF1	1.98	5.00E-05	SAMD8	1.65	5.00E-05	PYCR1	-1.57	5.00E-05
GATAD2A	-1.56	1.00E-04	AMBP	1.97	5.00E-05	TMEM87B	1.65	5.00E-05	VEGFB	-1.57	5.00E-05
MYPOP	-1.57	1.00E-04	LIFR	1.97	5.00E-05	TXNRD1	1.65	5.00E-05	KNSTRN	-1.58	5.00E-05
ANKRD2	-1.59	1.00E-04	TBC1D9	1.97	5.00E-05	VCPIP1	1.65	5.00E-05	LRRC8A	-1.58	5.00E-05
TBCD	-1.64	1.00E-04	DCUN1D1	1.96	5.00E-05	KBTBD6	1.64	5.00E-05	TPGS2	-1.58	5.00E-05
ADAM8	-1.66	1.00E-04	NAP1L2	1.96	5.00E-05	PELI2	1.64	5.00E-05	CDH4	-1.59	5.00E-05
GRB7	-1.66	1.00E-04	RNF38	1.96	5.00E-05	FRMD4B	1.63	5.00E-05	FAM64A	-1.59	5.00E-05
SMOC1	-1.67	1.00E-04	DNAJB4	1.95	5.00E-05	PRKAR1A	1.63	5.00E-05	KRT7	-1.59	5.00E-05
ACD	-1.69	1.00E-04	KLHL11	1.95	5.00E-05	FAM114A1	1.62	5.00E-05	ULK3	-1.59	5.00E-05
PDZK1IP1	-1.73	1.00E-04	HHIPL2	1.94	5.00E-05	KIAA0232	1.62	5.00E-05	CD276	-1.6	5.00E-05
DUSP7	-1.76	1.00E-04	MEX3B	1.93	5.00E-05	RASA2	1.62	5.00E-05	CYP2S1	-1.6	5.00E-05
KHK	-1.79	1.00E-04	SRGN	1.93	5.00E-05	N4BP2	1.61	5.00E-05	HNRPDL	-1.6	5.00E-05
RHOF	-1.8	1.00E-04	GCC2	1.92	5.00E-05	USP46	1.61	5.00E-05	KIF2C	-1.6	5.00E-05
ASIC3	-1.83	1.00E-04	GCLM	1.92	5.00E-05	AK3	1.6	5.00E-05	MFSD3	-1.6	5.00E-05
FANCA	-1.84	1.00E-04	ITGAV	1.92	5.00E-05	AMMECR1	1.6	5.00E-05	APOBEC3B	-1.61	5.00E-05
SLC25A19	-1.85	1.00E-04	XPR1	1.92	5.00E-05	DESI2	1.6	5.00E-05	C6orf1	-1.61	5.00E-05
MOB3B	-1.96	1.00E-04	MAP3K2	1.91	5.00E-05	SGCB	1.6	5.00E-05	FTSJ1	-1.61	5.00E-05
FAM167A	-2.03	1.00E-04	HINT3	1.9	5.00E-05	UBASH3B	1.6	5.00E-05	GINS3	-1.61	5.00E-05
TP63	-2.06	1.00E-04	HIPK3	1.9	5.00E-05	WDR31	1.6	5.00E-05	PAQR6	-1.61	5.00E-05
L3MBTL1	-2.08	1.00E-04	NR0B1	1.9	5.00E-05	APPBP2	1.59	5.00E-05	SEMA4B	-1.61	5.00E-05
SAMD4B	-2.15	1.00E-04	PPP1R2	1.9	5.00E-05	CCNT2	1.59	5.00E-05	XYLT2	-1.61	5.00E-05
CCAT1	-2.18	1.00E-04	NAMPT	1.89	5.00E-05	FAM129A	1.59	5.00E-05	CDCA8	-1.62	5.00E-05
CHTF18	-2.29	1.00E-04	43531	1.88	5.00E-05	GAN	1.59	5.00E-05	CHEK2	-1.62	5.00E-05
KCNJ16	-2.3	1.00E-04	RP2	1.88	5.00E-05	CCDC50	1.58	5.00E-05	RPL3	-1.62	5.00E-05
UCP3	-2.3	1.00E-04	UBE2H	1.88	5.00E-05	CCDC90B	1.58	5.00E-05	MAP2K3	-1.63	5.00E-05

(continued)

ID	logFC	P.Value	ID	logFC	P.Value	ID	logFC	P.Value	ID	logFC	P.Value
POLD2	-1.63	5.00E-05	DHFR	-1.91	5.00E-05	EPAS1	-2.24	5.00E-05	RUNX3	-2.85	5.00E-05
SMPD2	-1.63	5.00E-05	MAP1S	-1.91	5.00E-05	INSL4	-2.24	5.00E-05	SCARA5	-2.85	5.00E-05
DNAJC9	-1.64	5.00E-05	PSRC1	-1.91	5.00E-05	TTLL12	-2.25	5.00E-05	C21orf58	-2.86	5.00E-05
GHDC	-1.64	5.00E-05	ITGB4	-1.92	5.00E-05	ZNF300	-2.25	5.00E-05	MGAT5B	-2.87	5.00E-05
PTPN18	-1.64	5.00E-05	ORC1	-1.92	5.00E-05	HAS2	-2.26	5.00E-05	TRAIP	-2.88	5.00E-05
CDK1	-1.65	5.00E-05	RPL6P27	-1.92	5.00E-05	RAD51	-2.26	5.00E-05	WDR62	-2.91	5.00E-05
IL11	-1.65	5.00E-05	GLI2	-1.93	5.00E-05	IQGAP3	-2.27	5.00E-05	C1QL1	-2.95	5.00E-05
EFEMP1	-1.66	5.00E-05	REEP2	-1.93	5.00E-05	NCAPD2	-2.27	5.00E-05	MROH6	-2.95	5.00E-05
HELLS	-1.66	5.00E-05	RPL22L1	-1.93	5.00E-05	CDCA5	-2.28	5.00E-05	MANEAL	-3.04	5.00E-05
MGLL	-1.66	5.00E-05	C19orf24	-1.94	5.00E-05	COLGALT1	-2.28	5.00E-05	PHGDH	-3.04	5.00E-05
PDK4	-1.66	5.00E-05	EIF4EBP1	-1.95	5.00E-05	FBXO43	-2.28	5.00E-05	PCSK9	-3.06	5.00E-05
TMEM219	-1.66	5.00E-05	HJURP	-1.95	5.00E-05	UBE2C	-2.28	5.00E-05	POU5F1	-3.07	5.00E-05
TPX2	-1.66	5.00E-05	TUBB3	-1.95	5.00E-05	RMI2	-2.29	5.00E-05	E2F1	-3.09	5.00E-05
HSD3B7	-1.67	5.00E-05	ZFYVE19	-1.95	5.00E-05	WNT7B	-2.29	5.00E-05	RP11-726G1.1	-3.11	5.00E-05
IRAK1	-1.67	5.00E-05	PXN	-1.96	5.00E-05	PACSIN3	-2.3	5.00E-05	SLC6A16	-3.12	5.00E-05
NAGA	-1.67	5.00E-05	BRIP1	-1.97	5.00E-05	NUDC	-2.32	5.00E-05	C9orf16	-3.14	5.00E-05
TBC1D16	-1.67	5.00E-05	CEP128	-1.97	5.00E-05	C1orf159	-2.33	5.00E-05	IGFL4	-3.17	5.00E-05
TIMELESS	-1.67	5.00E-05	PC	-1.97	5.00E-05	KRT81	-2.33	5.00E-05	TROAP	-3.2	5.00E-05
AURKA	-1.68	5.00E-05	ANP32B	-1.98	5.00E-05	SALL4	-2.33	5.00E-05	PKMYT1	-3.21	5.00E-05
FOXP4	-1.68	5.00E-05	CYB5R3	-1.98	5.00E-05	ASF1B	-2.34	5.00E-05	C11orf24	-3.22	5.00E-05
HAUS7	-1.68	5.00E-05	FOXRED2	-1.98	5.00E-05	IMMP1L	-2.34	5.00E-05	RADIL	-3.23	5.00E-05
ITFG3	-1.68	5.00E-05	LINC00263	-1.98	5.00E-05	CLTCL1	-2.35	5.00E-05	TNFSF14	-3.23	5.00E-05
LINC00707	-1.68	5.00E-05	PLXND1	-1.98	5.00E-05	DDX11	-2.35	5.00E-05	C1orf233	-3.32	5.00E-05
LSR	-1.68	5.00E-05	NABP2	-1.99	5.00E-05	C11orf86	-2.37	5.00E-05	MYBL2	-3.33	5.00E-05
ORC6	-1.68	5.00E-05	NOP56	-1.99	5.00E-05	PADI1	-2.37	5.00E-05	GTSE1	-3.37	5.00E-05
POLA2	-1.68	5.00E-05	RECQL4	-1.99	5.00E-05	DEPDC1B	-2.39	5.00E-05	PIF1	-3.39	5.00E-05
RBBP8	-1.68	5.00E-05	SH3GL1	-1.99	5.00E-05	DMBX1	-2.39	5.00E-05	SLCO4A1	-3.39	5.00E-05
ATAD5	-1.69	5.00E-05	LMTK3	-2	5.00E-05	WDR76	-2.39	5.00E-05	KIF18B	-3.4	5.00E-05
SCRN2	-1.69	5.00E-05	C11orf84	-2.01	5.00E-05	GTF3C5	-2.41	5.00E-05	PAQR4	-3.4	5.00E-05
FAM195A	-1.7	5.00E-05	EPN1	-2.01	5.00E-05	TOX2	-2.41	5.00E-05	C19orf48	-3.41	5.00E-05
LRP5L	-1.7	5.00E-05	FGG	-2.01	5.00E-05	TRPC6	-2.41	5.00E-05	FGB	-3.42	5.00E-05
NCAPD3	-1.7	5.00E-05	IGFBP4	-2.01	5.00E-05	RHOV	-2.42	5.00E-05	NPR1	-3.42	5.00E-05
POLQ	-1.7	5.00E-05	KRT19	-2.01	5.00E-05	POLR2E	-2.43	5.00E-05	CDCA3	-3.49	5.00E-05
RIN1	-1.7	5.00E-05	MTFR2	-2.01	5.00E-05	NID1	-2.45	5.00E-05	RDM1	-3.49	5.00E-05
KATNB1	-1.71	5.00E-05	AP1M1	-2.02	5.00E-05	POLE	-2.45	5.00E-05	E2F2	-3.51	5.00E-05
SIRPA	-1.71	5.00E-05	DNMT1	-2.02	5.00E-05	SPOCD1	-2.45	5.00E-05	FOXM1	-3.54	5.00E-05
IFI6	-1.72	5.00E-05	PIP5K1C	-2.02	5.00E-05	CDCP1	-2.46	5.00E-05	C5AR1	-3.6	5.00E-05
LMNB2	-1.72	5.00E-05	RFC2	-2.02	5.00E-05	SKA1	-2.46	5.00E-05	C1orf61	-3.63	5.00E-05
NDOR1	-1.72	5.00E-05	TUBB	-2.02	5.00E-05	ZNF594	-2.46	5.00E-05	ZNF688	-3.74	5.00E-05
ARV1	-1.73	5.00E-05	UHRF1	-2.02	5.00E-05	FZD8	-2.47	5.00E-05	NPTX1	-3.81	5.00E-05
ATAD3A	-1.73	5.00E-05	GOLT1A	-2.03	5.00E-05	SREBF1	-2.47	5.00E-05	TERT	-3.85	5.00E-05
CLSTN3	-1.73	5.00E-05	NFKBID	-2.03	5.00E-05	GREM1	-2.48	5.00E-05	TGM2	-3.97	5.00E-05
MOGS	-1.73	5.00E-05	SFXN2	-2.03	5.00E-05	MRM1	-2.48	5.00E-05	SUSD2	-4.05	5.00E-05
RANGRF	-1.73	5.00E-05	BRCA1	-2.05	5.00E-05	STC1	-2.48	5.00E-05	ELN	-4.31	5.00E-05
TMEM237	-1.73	5.00E-05	C1orf116	-2.05	5.00E-05	TUBA1B	-2.48	5.00E-05	TNS4	-4.43	5.00E-05
TPI1	-1.73	5.00E-05	CALR	-2.05	5.00E-05	CDC25A	-2.49	5.00E-05	NCKAP5	-4.67	5.00E-05
CTSD	-1.74	5.00E-05	MPP2	-2.05	5.00E-05	MCM10	-2.49	5.00E-05			
MND1	-1.74	5.00E-05	MRPS34	-2.05	5.00E-05	RP4-564F22.2	-2.49	5.00E-05			
STRIP2	-1.74	5.00E-05	NCS1	-2.05	5.00E-05	AP006621.5	-2.5	5.00E-05			
CEP55	-1.75	5.00E-05	MOSPD3	-2.06	5.00E-05	IGFBP3	-2.5	5.00E-05			
CNPY3	-1.75	5.00E-05	RTTN	-2.06	5.00E-05	SRRM2	-2.5	5.00E-05			
MLF1IP	-1.75	5.00E-05	KCNK5	-2.07	5.00E-05	DPYSL5	-2.51	5.00E-05			
POP7	-1.75	5.00E-05	LIG1	-2.07	5.00E-05	DTL	-2.51	5.00E-05			
TRPM6	-1.75	5.00E-05	AVPI1	-2.08	5.00E-05	FGL1	-2.52	5.00E-05			
TUBG1	-1.75	5.00E-05	TUBB4B	-2.08	5.00E-05	DSCC1	-2.53	5.00E-05			
HENMT1	-1.77	5.00E-05	UQCR10	-2.08	5.00E-05	FANCD2	-2.53	5.00E-05			
SRM	-1.77	5.00E-05	CEP72	-2.09	5.00E-05	SLFNL1	-2.55	5.00E-05			
MCM4	-1.78	5.00E-05	CIT	-2.09	5.00E-05	CSRP1	-2.56	5.00E-05			
RCCD1	-1.78	5.00E-05	RRM2	-2.1	5.00E-05	CTC-360G5.1	-2.56	5.00E-05			
ARHGEF39	-1.79	5.00E-05	C16orf59	-2.11	5.00E-05	SULT2B1	-2.56	5.00E-05			
SHMT1	-1.79	5.00E-05	WDR54	-2.11	5.00E-05	PEMT	-2.58	5.00E-05			
AARS	-1.8	5.00E-05	NTS	-2.12	5.00E-05	PADI3	-2.59	5.00E-05			
AGFG2	-1.81	5.00E-05	SAMD1	-2.12	5.00E-05	AURKB	-2.61	5.00E-05			
MAP4	-1.81	5.00E-05	CABIN1	-2.13	5.00E-05	PRKCDBP	-2.61	5.00E-05			
MCM7	-1.81	5.00E-05	CDC20	-2.13	5.00E-05	SAPCD2	-2.61	5.00E-05			
PODXL2	-1.81	5.00E-05	CDT1	-2.13	5.00E-05	MMP15	-2.62	5.00E-05			
EXO1	-1.82	5.00E-05	CENPM	-2.13	5.00E-05	EFNB2	-2.63	5.00E-05			
PALM	-1.82	5.00E-05	COPZ2	-2.13	5.00E-05	C17orf53	-2.64	5.00E-05			
SAP130	-1.82	5.00E-05	CPS1	-2.13	5.00E-05	FANCG	-2.64	5.00E-05			
SKA3	-1.82	5.00E-05	HSPG2	-2.13	5.00E-05	IRF2BP1	-2.64	5.00E-05			
SLC29A4	-1.82	5.00E-05	POLA1	-2.13	5.00E-05	CABLES2	-2.65	5.00E-05			
ZNF331	-1.82	5.00E-05	SGK223	-2.13	5.00E-05	FST	-2.66	5.00E-05			
43525	-1.83	5.00E-05	SIGMAR1	-2.13	5.00E-05	PADI2	-2.66	5.00E-05			
DKK1	-1.83	5.00E-05	SPARC	-2.13	5.00E-05	RP11-420A23.1	-2.66	5.00E-05			
DSN1	-1.83	5.00E-05	KIF15	-2.14	5.00E-05	POLE2	-2.67	5.00E-05			
STRA13	-1.83	5.00E-05	KIFC1	-2.14	5.00E-05	REEP6	-2.68	5.00E-05			
KIF11	-1.84	5.00E-05	XRCC2	-2.14	5.00E-05	C1QL4	-2.69	5.00E-05			
NCAPH	-1.84	5.00E-05	AGPAT1	-2.15	5.00E-05	DIO2	-2.69	5.00E-05			
CDCA7L	-1.85	5.00E-05	MCM6	-2.15	5.00E-05	VKORC1	-2.69	5.00E-05			
GMNN	-1.85	5.00E-05	SFMBT2	-2.15	5.00E-05	CDC45	-2.7	5.00E-05			
POP5	-1.85	5.00E-05	CTSL2	-2.16	5.00E-05	DHRS11	-2.7	5.00E-05			
CDK2AP2	-1.86	5.00E-05	CKB	-2.17	5.00E-05	LCN2	-2.7	5.00E-05			
DDX39A	-1.86	5.00E-05	IFI44	-2.17	5.00E-05	MCM3	-2.71	5.00E-05			
KXD1	-1.86	5.00E-05	MCM5	-2.17	5.00E-05	MKI67	-2.72	5.00E-05			
TMEM107	-1.86	5.00E-05	43711	-2.18	5.00E-05	NME4	-2.72	5.00E-05			
FGA	-1.87	5.00E-05	CNFN	-2.18	5.00E-05	CALB2	-2.73	5.00E-05			
FHL2	-1.87	5.00E-05	FCHO1	-2.18	5.00E-05	TONSL	-2.73	5.00E-05			
GINS1	-1.87	5.00E-05	PFAS	-2.18	5.00E-05	PPP1R1C	-2.74	5.00E-05			
NUCB1	-1.87	5.00E-05	PTMA	-2.18	5.00E-05	IQCC	-2.75	5.00E-05			
ATAT1	-1.88	5.00E-05	SLC25A22	-2.18	5.00E-05	NR4A1	-2.77	5.00E-05			
CNKSR2	-1.88	5.00E-05	RNFT2	-2.19	5.00E-05	TICRR	-2.77	5.00E-05			
KIAA2013	-1.88	5.00E-05	MCM2	-2.2	5.00E-05	ARHGEF4	-2.78	5.00E-05			
PAK4	-1.88	5.00E-05	MSH5	-2.2	5.00E-05	TCOF1	-2.79	5.00E-05			
POLD1	-1.88	5.00E-05	NMB	-2.2	5.00E-05	FAM111B	-2.81	5.00E-05			
PXDN	-1.88	5.00E-05	SLC29A2	-2.21	5.00E-05	FASN	-2.82	5.00E-05			
KCTD17	-1.89	5.00E-05	C6orf141	-2.22	5.00E-05	CHCHD10	-2.83	5.00E-05			
RAB3IL1	-1.89	5.00E-05	CDC6	-2.22	5.00E-05	B3GNT3	-2.84	5.00E-05			
AREG	-1.9	5.00E-05	NES	-2.22	5.00E-05	ESPL1	-2.84	5.00E-05			
DBF4B	-1.9	5.00E-05	TK1	-2.23	5.00E-05	RBPMS2	-2.84	5.00E-05			
NENF	-1.9	5.00E-05	ZNF488	-2.23	5.00E-05	CLSPN	-2.85	5.00E-05			

Table S2. PCR primers used in this study

Target	Sequence (5'→3')
FAM188B	Forward : TTCCCCATCTGGGTGGTT
	Reverse : CAATGGTCAGCCGGATCT
FOXM1	Forward : ATCTCAAGCACCACTCCCTTG
	Reverse : CTTGCTGAGGCTGTCATTCA
β-catenin	Forward : TCCCACTAATGTCCAGCGTT
	Reverse : ATGGACCATAACTGCAGCCT
c-myc	Forward : TCAAGAGGCGAACACACAAC
	Reverse : TTTCCGCAACAAGTCCTCTT
GAPDH	Forward : TGCACCACCAACTGCTTA
	Reverse : GGATGCAGGGATGATGTTC

Table S3. Summary of immunohistochemistry analyses of FAM188B, beta-catenin, and FOXM1 with human lung tumor tissue microarray.

Tumor tissues	FAM188B	β-catenin	FOXM1
Positive	28 (60.9%)	46 (100%)	38 (82.6%)
Negative	18 (39.1%)	0 (0.0%)	8 (17.4%)
No. of tumor tissues	46 (100%)	46 (100%)	46 (100%)

Normal tissues	FAM188B	β-catenin	FOXM1
Positive	0 (0.0%)	0 (0.0%)	0 (0.0%)
Negative	9 (100%)	9 (100%)	9 (100%)
No. of normal tissues	9 (100%)	9 (100%)	9 (100%)

Table S4. Immunohistochemistry score for the entire tissue microarray of lung tumors and normal lung tissues used in this study.

Arrangement of TMA	Lung tumor 1	Lung tumor 2	Lung tumor 3	Lung tumor 4	Lung tumor 5	Lung tumor 6	Lung tumor 7	Lung tumor 8	Lung tumor 9	Lung tumor 10
	Lung tumor 11	Lung tumor 12	Lung tumor 13	Lung tumor 14	Lung tumor 15	Lung tumor 16	Lung tumor 17	Lung tumor 18	Lung tumor 19	Lung tumor 20
	Lung tumor 21	Lung tumor 22	Lung tumor 23	Lung tumor 24	Lung tumor 25	Lung tumor 26	Lung tumor 27	Lung tumor 28	Lung tumor 29	Lung tumor 30
	Lung tumor 31	Lung tumor 32	Lung tumor 33	Lung tumor 34	Lung tumor 35	Lung tumor 36	Lung tumor 37	Lung tumor 38	Lung tumor 39	Lung tumor 40
	Lung tumor 41	Lung tumor 42	Lung tumor 43	Lung tumor 44	Lung tumor 45	Lung tumor 46	Lung tumor 47	Lung tumor 48	Lung tumor 49	Lung tumor 50
	Normal lung 1	Normal lung 2	Normal lung 3	Normal lung 4	Normal lung 5	Normal lung 6	Normal lung 7	Normal lung 8	Normal lung 9	Carbon (-) control

b-catenin	M 3	M 1	M 2	M 3	M 2	M 3	M 2	M 2	M 2	M 3
	N 3 25	M 2	M 3	M 3	M 2	M 3	M 3	M 2	M 2	M 2
	No tissue	N 3	M 3	M 2	M 2	M 1	M 1	M 1	No tumor	M 1
	M 2	M 3	M 2	M 2	M 2	M 1	M 2	M 2	M 3	M 3
	M 1	M 2	M 1	No tissue	M 3	M 1	M 3	M 1	M 3	M 3
	-	-	-	-	-	-	-	-	-	●

FOXM1	-	-	N1 10	C1	N1 40/C1 10	-	C1 30	N2 20	N2 20	-
	C 2	C 1	C 2	C1	C1	C1	C2	C2	C1 40	N2 30
	No tissue	N1 20/C 120	N1 5	-	C1	C1 30	-	No tissue	No tumor	-
	N1 20/C130	C1	N1 20/C1 50	N2 40/C1 60	C1	-	C1	C1	C1	N1 20/C1 20
	N3 10/C2 20	N2 30	C1 20	No tissue	C1	N2 30/C1 30	C2	N1 5	C1	N3 30
	-	-	-	-	-	-	-	-	-	●

FAM188B Cytoplasm	-	-	N1 20	-	-	1	-	1	1	-
	1	-	2	1	1	1	1	1	C1 30	2
	No tissue	-	1 5	-	1	1 20	3 25	No tissue	No tumor	-
	1	-	-	N1 30/C1 40	-	-	-	1	1	1
	N 30	1	-	No tissue	1	-	1	N2 5/C1 5	-	C1 20
	-	-	-	-	-	-	-	-	-	●

N: nucleus
C: Cytoplasm
M: membranous
intensity 1 - 3
(+) cell percent
